# Multi-Criteria Decision Analysis using Physician Preferences from a Global Discrete Choice Experiment to Investigate the Selection of Preemptive Antiviral Treatments for Post-Hematopoietic Cell Transplant Cytomegalovirus Infection

**DOI:** 10.1093/ofid/ofag500

**Published:** 2026-09-01

**Authors:** Miguel-Angel Perales, Jo-Anne H Young, Tommi Tervonen, Stephane Deparis, Jonathan D Norton, Cristian Gugiu, Tien Bo, Kimberly Davis, ShunPing Quan, Victoria L Prince, Adel Abou Ali, Jessica Auciello

**Affiliations:** Adult Bone Marrow Transplantation Service, Department of Medicine, Memorial Sloan Kettering Cancer Center, New York, New York, USA; Department of Medicine, Weill Cornell Medical College, New York, New York, USA; Department of Medicine, University of Minnesota, Minneapolis, Minnesota, USA; Kielo Research, Zug, Switzerland; Kielo Research, Zug, Switzerland; Takeda Development Center Americas, Inc., Cambridge, Massachusetts, USA; Takeda Development Center Americas, Inc., Cambridge, Massachusetts, USA; Takeda Development Center Americas, Inc., Cambridge, Massachusetts, USA; Takeda Development Center Americas, Inc., Cambridge, Massachusetts, USA; Takeda Development Center Americas, Inc., Cambridge, Massachusetts, USA; Takeda Development Center Americas, Inc., Cambridge, Massachusetts, USA; Takeda Development Center Americas, Inc., Cambridge, Massachusetts, USA; Takeda Development Center Americas, Inc., Cambridge, Massachusetts, USA

**Keywords:** cytomegalovirus, discrete choice experiment, hematopoietic cell transplant, multi-criteria decision analysis, physician treatment preference

## Abstract

**Background:**

A discrete choice experiment (DCE) subsequently incorporated into a multi-criteria decision analysis (MCDA) was used to compare clinicians’ trade-offs between the benefits and risks of preemptive anti-cytomegalovirus (CMV) treatments posthematopoietic cell transplant (HCT).

**Methods:**

In the DCE, physicians completed forced-choice tasks comparing hypothetical treatments. We used logit models to estimate preference weights, relative attribute importance (RAI) score, and minimum acceptable benefit. The MCDA combined DCE-derived weighted attributes with clinical performance data to compare maribavir versus valganciclovir (using data from the AURORA study) and maribavir versus valganciclovir, ganciclovir, and foscarnet (using data from multiple sources). Analyses were conducted for 2 cohorts defined by baseline absolute neutrophil count (ANC) <1500/mm^3^ or ≥1500/mm^3^ at CMV reactivation.

**Results:**

In the DCE, all 7 attributes significantly impacted physician (n = 377) treatment choices; the most important were administration route/monitoring (RAI score = 25.7%), nephrotoxicity risk (RAI score = 18.5%), grade 3–4 neutropenia risk (RAI score = 18.1%), and week 8 viremia clearance (RAI score = 16.0%). Neutropenia at CMV reactivation significantly reduced physicians’ preference for treatments with a higher grade 3–4 neutropenia risk. In the MCDA, overall benefit–risk scores were higher for maribavir than valganciclovir in both ANC cohorts. Among the considered attributes, the largest positive contributor to the overall benefit–risk score was viremia clearance at week 8, and the largest negative contributor was grade 3–4 neutropenia risk. Maribavir had a higher overall benefit–risk score than ganciclovir and foscarnet in both ANC cohorts.

**Conclusions:**

Within this MCDA framework, maribavir was preferred over valganciclovir, ganciclovir, and foscarnet for preemptive treatment of CMV after HCT.

## Teaser

Within this discrete choice experiment–informed multi-criteria decision analysis (MCDA), physician preference for maribavir treatment for posthematopoietic cell transplant (HCT) cytomegalovirus was driven by oral administration versus ganciclovir and foscarnet, and a lower risk of neutropenia and nephrotoxicity versus valganciclovir, ganciclovir, and foscarnet. Elevator pitch required (limit 20 000 characters) for submission site only, as per the following guidance: “To help us better understand and highlight the significance of your work, we kindly ask that you include a brief ‘elevator pitch’ with your manuscript submission. In 3–4 sentences, please describe: ° The key impact of your study. ° The gap in knowledge it addresses. ° How it could influence clinical practice, healthcare policy, or infectious disease research.” This study quantifies how physicians trade-off efficacy, toxicity, and administration when selecting preemptive CMV therapy after HCT by utilizing an MCDA informed by a global discrete choice experiment. It addresses a gap in evidence on which treatment attributes most drive clinician choice, including in patients with versus without neutropenia at CMV reactivation. Physicians prioritized oral administration and avoiding grade 3–4 neutropenia and nephrotoxicity over incremental differences in CMV viremia clearance. In the MCDA, maribavir had higher overall benefit–risk scores than valganciclovir, ganciclovir, and foscarnet in patients with and without neutropenia at baseline.

## Graphical Abstract

**Graphical Abstract:**
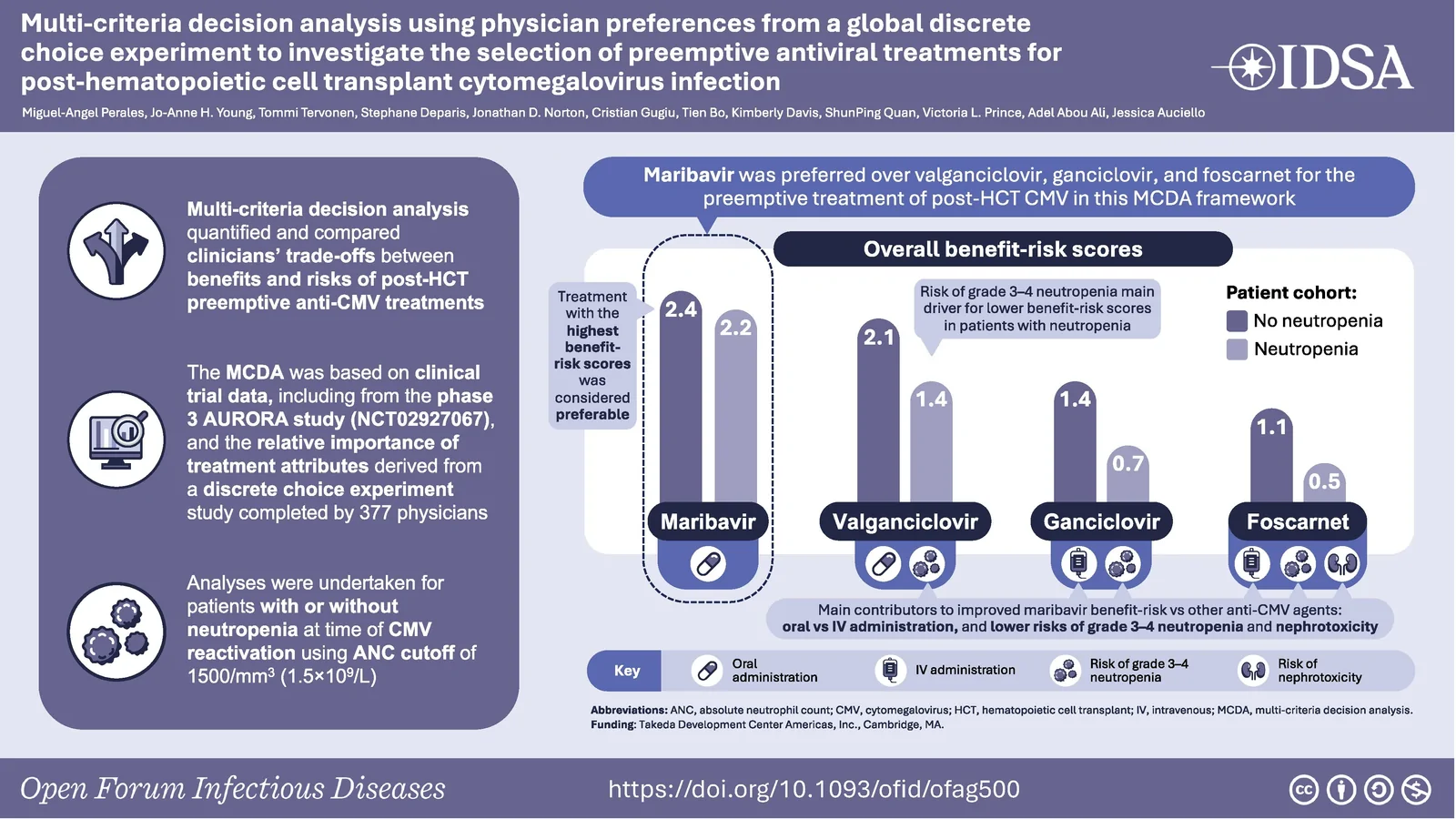

Cytomegalovirus (CMV) infection in hematopoietic cell transplant (HCT) recipients is managed by 2 complementary standard practices: prophylaxis and/or preemptive treatment [1–3]. In preemptive treatment, patients are typically monitored for CMV viral load once weekly, and antiviral treatment initiated when viremia is detected at a prespecified threshold and discontinued when viremia is cleared [1–3].

Several antiviral agents are available for CMV treatment, of which the most commonly used are ganciclovir and its oral prodrug valganciclovir [1, 2]. However, these 2 agents are often associated with myelosuppression [1, 4, 5]. In cases where neutropenia already exists, foscarnet is used, although foscarnet has limitations: it is only available as an intravenous formulation, and is associated with nephrotoxicity and electrolyte depletion [1, 5, 6].

Maribavir was approved by the US Food and Drug Administration in 2021, as well as in certain other countries, as an oral treatment for refractory (with or without resistance) posttransplant CMV infection/disease or for intolerance to other anti-CMV therapies [7–9]. In the phase 3, randomized, open-label SOLSTICE study (NCT02931539), maribavir showed superiority over investigator-assigned therapy (ganciclovir, valganciclovir, foscarnet, or cidofovir) for CMV viremia clearance at week 8 in HCT and solid-organ transplant recipients with refractory CMV infection [10]. Maribavir has an improved safety profile versus valganciclovir/ganciclovir (neutropenia) and foscarnet (acute kidney injury) [10]. Subsequently, the phase 3, randomized, double-blind AURORA study (NCT02927067) compared the efficacy and safety of maribavir with valganciclovir for preemptive treatment of the first asymptomatic CMV infection after HCT [11]. Noninferiority of maribavir versus valganciclovir was not met for the primary endpoint of confirmed CMV viremia clearance at week 8. Maribavir demonstrated similar CMV viremia clearance with no clinical findings of CMV tissue-invasive disease during the posttreatment follow-up (at weeks 12, 16, and 20) and an improved tolerability profile, with fewer discontinuations resulting from neutropenia [11].

Selection of preemptive treatment for CMV infection after HCT requires consideration of several aspects, including the efficacy and safety profiles of anti-CMV agents, as well as host conditions at the time of transplant or therapy initiation. An online survey including a discrete choice experiment (DCE) was used to assess physician preferences for attributes relevant to preemptive treatment choice for post-HCT CMV infection. A DCE is routinely applied to predict patient and clinician preferences for treatments and health services [12] and has been shown to have strong predictive accuracy compared with preferences collected via other methods [13].

We then applied a multi-criteria decision analysis (MCDA) to quantify and compare benefit–risk trade-offs across antiviral options (maribavir, valganciclovir, ganciclovir, and foscarnet). An MCDA is a structured, quantitative approach that integrates evidence across several outcomes by weighting each attribute according to its relative importance to stakeholders and then combining these weighted values into an overall performance score for each intervention [14, 15]. In this study, clinical trial data for each antiviral option were mapped against the corresponding DCE-derived attribute weight, and the attribute-specific values multiplied by the corresponding weight. These values were then combined to yield an aggregate score representing the overall benefit–risk profile from the respondent's perspective. This analysis approach has been previously used to support decision-making in the healthcare community [14, 15], including evaluating physician treatment preference and identification of attributes that determine this selection [16]. Indeed, the Professional Society for Health Economics and Outcomes Research has included MCDA within their good practice guidelines for quantitative benefit–risk assessment [17].

## METHODS

### DCE Survey Overview

We conducted a self-administered web-based survey including consent, screening, an introduction to attributes, DCE choice tasks, and a demographics questionnaire. The DCE was pretested in cognitive pilot interviews, followed by a soft launch of the survey with a subset of physicians to ensure the trade-offs were captured correctly (Supplementary Methods). The survey was piloted and administered in local languages. The soft launch and main survey data collection were completed between 6 September and 22 October 2024.

The study was conducted in compliance with applicable local regulations and was determined to be exempt by the Pearl Institutional Review Board (Indianapolis, IN, USA). All participants provided informed consent before being directed to the survey. Respondents were not informed that the study was associated with Takeda.

### Participants and Recruitment

Physicians were recruited in Australia, Canada, France, Germany, Italy, Spain, the United Kingdom, and the United States through a professional healthcare survey panel. Eligible physicians (hematology, oncology, HCT, or infectious disease specialists) had ≥2 years’ experience and had treated post-HCT CMV within the prior 12 months. Participants were remunerated for participating in the study after they had fully completed either the cognitive pilot interview or the survey.

### DCE Methodology and Statistical Analyses

The DCE was used to elicit physicians’ preferences for changes across attribute levels that were identified from a literature review (Supplementary Methods; Supplementary Tables 1 and 2). In each forced-choice task, participants selected between 2 experimentally designed treatments; therefore, respondents did not choose between actual treatments nor was any specific anti-CMV antiviral mentioned. Each task also included a patient profile describing CMV prophylaxis for 100 days prior to CMV reactivation (yes/no) and neutropenia at CMV reactivation (absolute neutrophil count [ANC] <1500/mm^3^ or ≥1500/mm^3^). The patient profile in relation to neutropenia was designed to change the interpretation of the grade 3–4 neutropenia risk attribute. For patients without neutropenia, this attribute was explained to be the risk of developing a new grade 3–4 neutropenia, whereas for patients with neutropenia, it represented the risk of worsening to a grade 3–4 neutropenia. No opt-out option was included in the design, as the scenario assumed CMV viremia reactivation at a level that necessitated treatment. Physicians completed 15 experimental choice tasks in addition to 2 nonexperimental tasks (ie, not used in estimation) to test the internal validity of the participants’ responses. Further details on the forced-choice task, including randomization and data quality variables, are provided in Supplementary Methods, Supplementary Table 3, and Supplementary Figure 1. The study sample size was chosen to enable meaningful quantitative insights into the preferences of the target population and to allow for the estimation of preferences sufficiently and precisely in key subgroups. The survey required responses to proceed; only complete surveys were analyzed, and thus there were no missing values. For the interpretation of *P*-values, *P* < .05 was considered statistically significant.

Descriptive analyses were conducted for all collected nonpreference data. Sociodemographic characteristics were analyzed by country, and data quality was analyzed by country, by specialization (infectious disease specialist vs others), and by years of experience (up to 10 years vs more than 10 years). Subgroup differences in categorical attributes were assessed using χ^2^ or Fisher's exact test. Significant differences in continuous variables were determined by Wilcoxon rank sum or Welch *t*-test. No adjustments for multiple comparisons were performed.

The DCE choices were analyzed within the Random Utility Theory framework [18–20], as described in the Supplementary Methods and Supplementary Table 4. Linearity of preferences was tested for all attributes except administration and monitoring using linear models and *R*^2^ assessment. All attributes showed linearity with *R*^2^ > 0.8 (lowest: 0.88 for dysgeusia risk); therefore, linear coding was applied in further analyses except for administration and monitoring (Supplementary Methods; Supplementary Table 5). Country pooling was assessed using a heteroscedastic multinomial logit (HMNL) model; scale heterogeneity was only observed in Australia versus the United States (Supplementary Table 6). Given the low number of participants in Australia, the scale differences were deemed acceptable, and data from all countries were pooled without adjusting for scale differences in further analyses.

A linear-coded model was used for base case analysis, from which the output measures of relative attribute importance (RAI) score (Supplementary Methods) and minimum acceptable benefit (MAB) were calculated. The RAI score quantifies the attribute influence; the higher the score, the more the attribute influenced the physicians’ choice versus other attributes. The MAB measures physician trade-offs against week 8 viremia clearance (ie, the minimum improvement in week 8 viremia clearance they require to accept a less desirable level in another attribute). The Krinsky–Robb procedure with 10 000 iterations was used to compute the 95% confidence intervals (CIs) around the MAB values and RAI scores [21].

Observed preference heterogeneity (ie, the variation in preference across individuals, where different people may value different aspects of a choice or decision situation, and these variations can be linked to attributes such as age, income, or other demographics) was assessed in subgroups (country, specialization, and years of experience) using logit models with an interaction term.

### Multi-Criteria Decision Analysis

The MCDA model incorporated treatment attributes of efficacy, safety, tolerability, and administration considerations that were identified from a targeted literature review. The MCDA used an additive value model combining DCE-derived attribute weights (Supplementary Table 7) with treatment performance data for each attribute (Supplementary Table 8) to generate benefit–risk scores and predicted choice probabilities, which express how likely, on average, a treatment is to be chosen from a set of options considered by a physician (Supplementary Methods).

Treatment attributes were analyzed in 3 separate analysis scenarios. The first 2 scenarios assessed maribavir versus valganciclovir based on data from the phase 3 AURORA trial, with the second scenario excluding the secondary endpoint of viremia clearance at 16 weeks (ie, 8 weeks of treatment and 8 weeks off treatment) [11] (Supplementary Table 7). This analysis was undertaken to avoid potential overrepresentation of the viremia clearance attribute, as the week 16 endpoint included patients who achieved viremia clearance at week 8 and then maintained clearance for a further 8 weeks off treatment. The third scenario assessed maribavir versus valganciclovir versus ganciclovir versus foscarnet based on multiple data sources (Supplementary Table 8). A probabilistic sensitivity analysis was also undertaken for the first scenario, employing bootstrapping to both clinical and preference uncertainty in estimating the benefit–risk scores and CIs (Supplementary Methods).

The MCDA was undertaken for 2 patient cohorts based on standard definitions of neutropenia (neutropenia: ANC < 1500/mm^3^, no neutropenia: ANC ≥ 1500/mm^3^) at the time of CMV reactivation [22].

## RESULTS

### Discrete Choice Experiment

#### Participant Disposition and Characteristics

Of 10 770 invited physicians, 377 completed the survey and were included in the analysis (Supplementary Figure 2), excluding 2 participants who straight lined by choosing treatment A in all choice tasks (Supplementary Table 3). Participant characteristics, including use of prophylactic agents, are summarized in Table 1 and Supplementary Tables 9 and 10. Overall, 60% of participants specialized in hematology, 41% in oncology, 40% in HCT management, and/or 31% in infectious disease management; participants were permitted to select >1 specialty. Most participants had ≥4 years of experience treating patients with post-HCT CMV infection. Most physicians worked in hospitals that had strict guidelines dictating treatment selection or flexible guidelines allowing physicians to select the treatment.

**Table 1.** Participant Demographics and Characteristics in the Discrete Choice Experiment

Characteristic	Overall N = 377
Age, years	
Mean (SD)	46.6 (8.6)
Median (range)	46 (28–76)
Sex, n (%)	
Male	272 (72.1)
Female	105 (27.9)
Years of experience treating patients with CMV infections post-HCT, n (%)	
More than 2, up to 3	15 (4.0)
More than 3, up to 4	24 (6.4)
4 or more	338 (89.7)
Years managing HCT patients, n (%)	
0–5	41 (10.9)
6–10	94 (24.9)
11–20	165 (43.8)
>20	77 (20.4)
Number of HCT patients seen on average per month, n (%)	
<2	34 (9.0)
3–5	78 (20.7)
6–10	107 (28.4)
>10	158 (41.9)
Are there guidelines on post-HCT CMV management where you work, n (%)	
Yes, the guidelines dictate which treatments need to be used	154 (40.8)
Yes, but I can choose any treatment for CMV	192 (50.9)
No	31 (8.2)
Specialty, n (%)^a^	
Hematology	228 (60.5)
Oncology	156 (41.4)
Infectious disease specialist	118 (31.3)
HCT specialist	149 (39.5)

Abbreviations: CMV, cytomegalovirus; HCT, hematopoietic cell transplant.

^a^Participants were permitted to select >1 specialty.

### Physician Preferences

**Figure 1.**
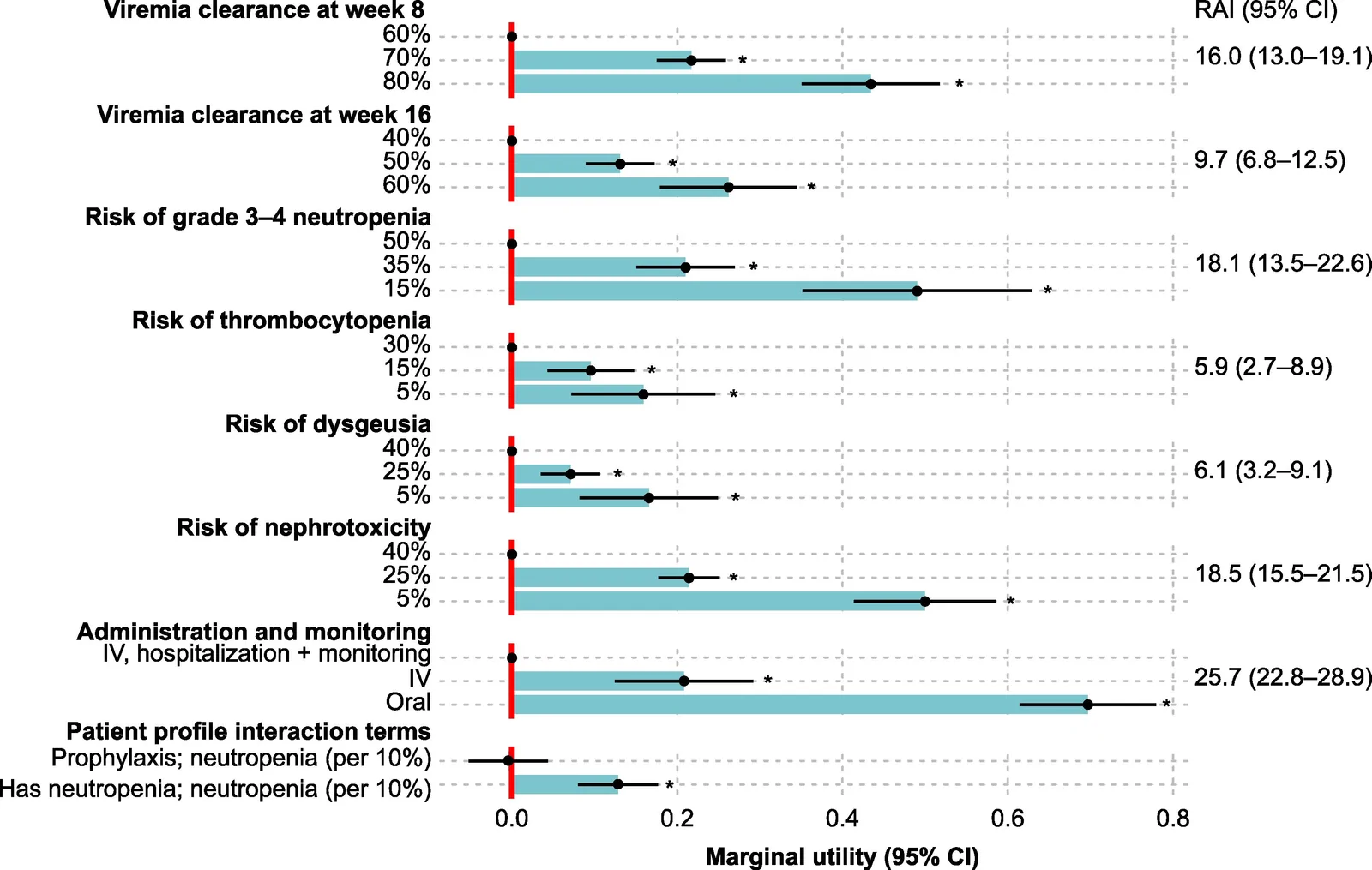
Linear-coded model of physician preferences with corresponding relative attribute importance scores from the discrete choice experiment. *P*  *<* .05 was considered statistically significant. *P < .001. IV, intravenous; RAI, relative attribute importance. RAI score (%) quantifies the influence of attributes; the higher the score, the more the attribute influenced the physicians’ choice versus other attributes. The marginal utility of a treatment attribute level is defined as the change in utility associated with moving from the reference level to that level, holding all other treatment attributes constant. Reprinted by permission from Springer Nature Limited: Bone Marrow Transplantation (2025) Abstracts Collection: The 51st Annual Meeting of the European Society for Blood and Marrow Transplantation: Physicians – Poster Session (P001-P909), 60:183-887; Perales MA, Tervonen T, Deparis S, et al. P530: Insights From a Discrete Choice Experiment: How Benefits and Risks Affect Physician Preferences for Pre-Emptive CMV Treatment in Hematopoietic Cell Transplant Recipients. Copyright 2025.

Physician preferences from the linear-coded model, together with the RAI scores, are presented in Figure 1. Route of administration/monitoring was the most influential attribute with oral route being preferred over IV with hospitalization/monitoring (RAI score = 25.7%), followed by nephrotoxicity risk, grade 3–4 neutropenia risk, and week 8 clearance. Prophylaxis use before CMV reactivation had no significant impact on physicians’ treatment preferences. In contrast, the presence or absence of neutropenia at CMV reactivation had a significant impact on physician preferences for avoiding the risk of grade 3–4 neutropenia. Physicians were more averse to the risk of neutropenia worsening to grade 3–4 versus the risk of patients without neutropenia developing grade 3–4 neutropenia.

The MAB analyses indicated physicians required at least a 32.1% increase in week 8 clearance to accept IV administration requiring hospitalization versus oral therapy, and a 6.5% or 6.6% increase to accept a 10% increase in nephrotoxicity or grade 3–4 neutropenia, respectively (Supplementary Table 11).

Preference heterogeneity by country, specialty, and experience was generally limited; no differences were observed between infectious disease specialists and other specialties, and data quality did not differ by specialty or experience (Supplementary Figures 3 and 4). Country-level differences are reported in Supplementary Results and Supplementary Figure 5.

### Multi-Criteria Decision Analysis

#### Maribavir Versus Valganciclovir (Primary Analysis)

**Figure 2.**
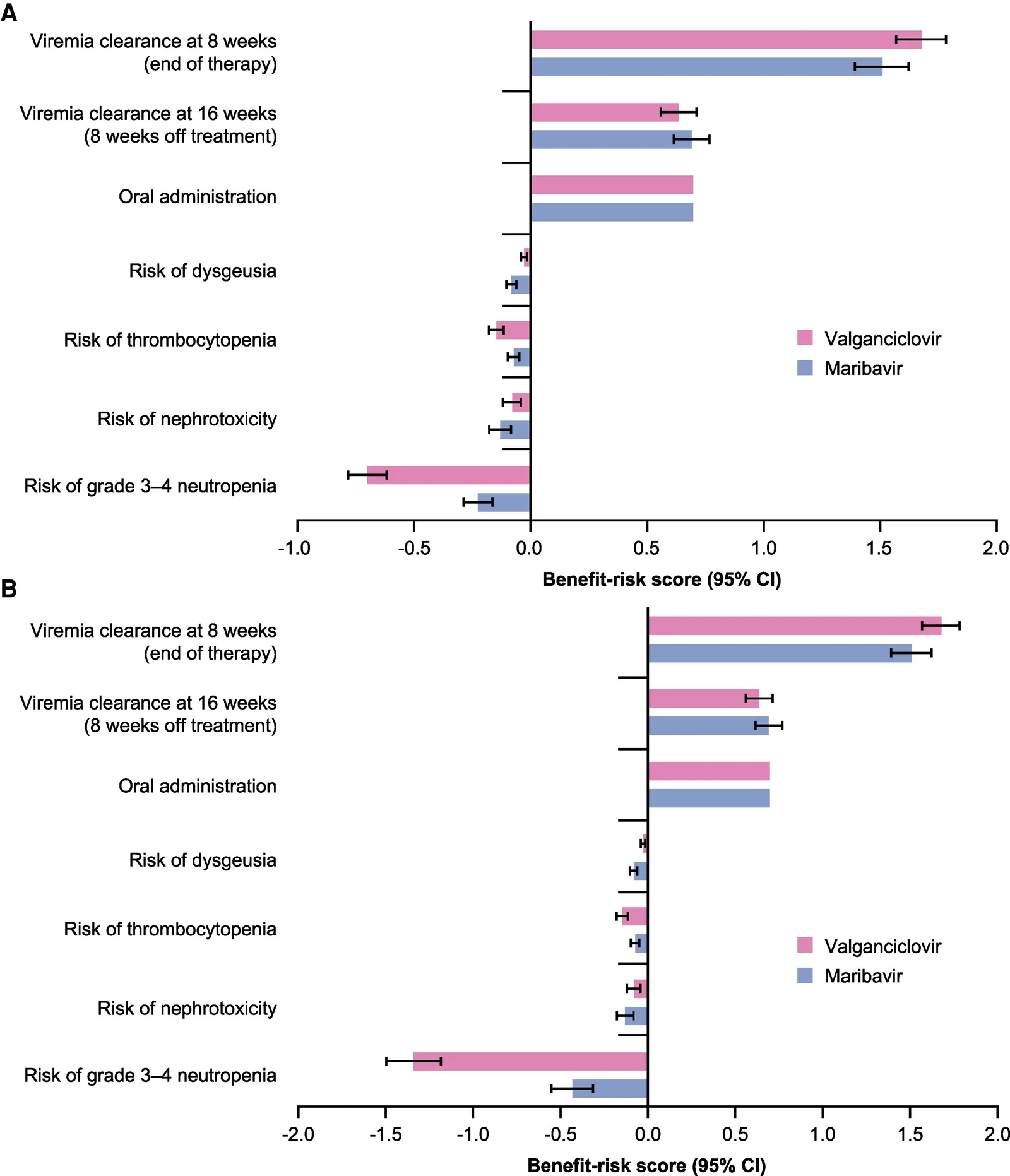
Attribute-level contributions to the benefit–risk score for maribavir and valganciclovir in patients with baseline absolute neutrophil count (*A*) ≥1500/mm^3^ (1.5 × 10^9^/L) and (*B*) <1500/mm^3^ (1.5 × 10^9^/L) in the multi-criteria decision analysis. The length of each bar is the product of the DCE-derived normalized attribute weight and standardized clinical trial data for the corresponding endpoint. 95% CI, 95% confidence interval.

**Figure 3.**
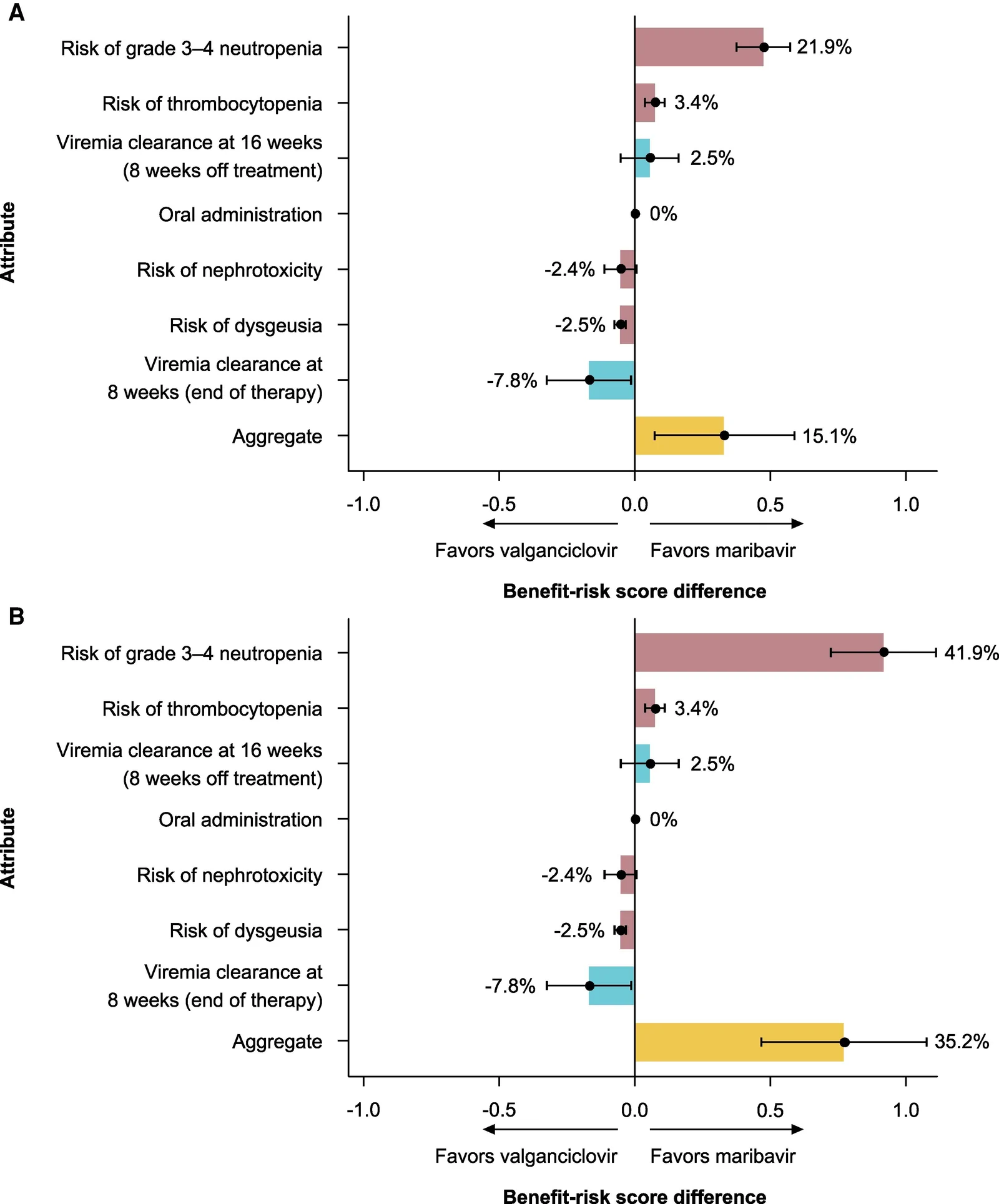
Benefit–risk score difference between maribavir and valganciclovir in patients with baseline ANC (*A*) ≥1500/mm^3^ (1.5 × 10^9^/L) and (*B*) <1500/mm^3^ (1.5 × 10^9^/L) in the multi-criteria decision analysis. The percentages displayed next to each bar represent changes in viremia clearance at week 8 (end of therapy) equivalent to the corresponding difference in benefit–risk scores shown on the *x*-axis. For example, in patients without baseline neutropenia (*A*), the lower risk of grade 3–4 neutropenia on maribavir has the same value as a 21.9% increase in week 8 viremia clearance, whereas in both figures week 8 viremia clearance is 7.8% lower with maribavir than with valganciclovir, so the value shown is −7.8% equivalent to itself. ANC, absolute neutrophil count. Reprinted by permission from Springer Nature Limited: Bone Marrow Transplantation (2025) Abstracts Collection: The 51st Annual Meeting of the European Society for Blood and Marrow Transplantation: Physicians – Poster Session (P001-P909), 60:183-887; Perales MA, Tervonen T, Deparis S, et al. P477: Implied Physician Preferences for Maribavir vs Valganciclovir for Treatment of Post-HCT CMV: Results From a Multi-Criteria Decision Analysis. Copyright 2025.

Maribavir had higher overall benefit–risk scores than valganciclovir in both ANC cohorts (Table 2). For both cohorts, viremia clearance at 8 weeks (end of therapy) was the largest positive contributor to the overall benefit–risk scores, with a slightly larger contribution for valganciclovir versus maribavir (Figure 2). Risk of grade 3–4 neutropenia was the largest negative contributor in both cohorts, with a larger magnitude of contribution for valganciclovir versus maribavir. Lower grade 3–4 neutropenia risk was the main driver of the score difference favoring maribavir, particularly in the ANC < 1500/mm^3^ cohort (Figure 3). Overall, the value difference between maribavir and valganciclovir for patients with baseline ANC ≥ 1500/mm^3^ and those with ANC < 1500/mm^3^ was equivalent to a 15.1% and 35.2% increase in viremia clearance at week 8, respectively. The predicted choice probabilities of maribavir versus valganciclovir for each of the 2 cohorts are reported in Table 2.

**Table 2.** **Overall Benefit–Risk Scores and Predicted Choice Probabilities in Patients With Baseline ANC**  **<**  **1500/mm^3^ (1.5 × 10^9^/L) and ≥1500/mm^3^ (1.5 × 10^9^/L) in the Multi**-**Criteria Decision Analysis**

Maribavir vs Valganciclovir		
	Maribavir	Valganciclovir
Baseline ANC ≥ 1500/mm^3^ (1.5 × 10^9^/L)		
Benefit–risk score (95% CI)	2.388 (2.197–2.576)	2.060 (1.871–2.240)
Benefit–risk score difference vs valganciclovir (95% CI)	0.328 (.07–.592)	…
Predicted choice probabilities^a^ (95% CI)	0.581 (.545–.617)	…
Baseline ANC < 1500/mm^3^ (1.5 × 10^9^/L)		
Benefit–risk score (95% CI)	2.182 (1.965–2.391)	1.419 (1.191–1.632)
Benefit–risk score difference vs valganciclovir (95% CI)	0.763 (.458–1.071)	…
Predicted choice probabilities^a^ (95% CI)	0.682 (.648–.713)	…

Maribavir vs valganciclovir including only week 8 viremia clearance		
	Maribavir	Valganciclovir
Baseline ANC ≥ 1500/mm^3^ (1.5 × 10^9^/L)		
Benefit–risk score (95% CI)	1.697 (1.562–1.832)	1.424 (1.275–1.567)
Benefit–risk score difference vs valganciclovir (95% CI)	0.273 (.077–.471)	…
Predicted choice probabilities^a^ (95% CI)	0.568 (.531–.605)	…
Baseline ANC < 1500/mm^3^ (1.5 × 10^9^/L)		
Benefit–risk score (95% CI)	1.490 (1.323–1.658)	0.783 (.585–.977)
Benefit–risk score difference vs valganciclovir (95% CI)	0.708 (.45–.967)	…
Predicted choice probabilities^a^ (95% CI)	0.670 (.637–.701)	…

Maribavir vs valganciclovir vs ganciclovir vs foscarnet				
	Maribavir	Valganciclovir	Ganciclovir	Foscarnet
Baseline ANC ≥ 1500/mm^3^ (1.5 × 10^9^/L)				
Benefit–risk score	2.372	2.051	1.354	1.109
Predicted choice probabilities^a^ (95% CI)	0.422 (.382–.462)	0.306 (.282–.33)	0.153 (.139–0.167)	0.119 (.107–.133)
Baseline ANC < 1500/mm^3^ (1.5 × 10^9^/L)				
Benefit–risk score	2.165	1.410	0.713	0.481
Predicted choice probabilities^a^ (95% CI)	0.529 (.489–.569)	0.249 (.227–.271)	0.124 (.111–.137)	0.098 (.086–.112)

Abbreviations: ANC, absolute neutrophil count; CI, confidence interval.

^a^Predicted choice probability expresses how likely, on average, a treatment is to be chosen from a set of options considered by a physician.

The probabilistic sensitivity analysis generated similar results although the CIs for the benefit–risk scores were wider than the primary analysis (Supplementary Table 12 and Supplementary Figure 6).

### Maribavir Versus Valganciclovir Including Only Week 8 Viremia Clearance

Results were consistent when week 16 clearance was excluded (Table 2). The overall value difference between maribavir and valganciclovir for patients with baseline ANC ≥ 1500/mm^3^ and those with ANC < 1500/mm^3^ was equivalent to a 12.6% and 32.6% increase in viremia clearance at week 8, respectively. The predicted choice probability of maribavir versus valganciclovir for each of the 2 cohorts is reported in Table 2.

### Maribavir Versus Valganciclovir Versus Ganciclovir Versus Foscarnet

In a comparison between 4 agents, maribavir had the highest benefit–risk scores in both ANC cohorts (Table 2 and Supplementary Figure 7), based on a slightly modified set of performance data (Supplementary Table 8). The largest negative contributors to the overall benefit–risk score for both cohorts were the risk of grade 3–4 neutropenia (all 4 agents) followed by risk of nephrotoxicity (foscarnet only; Supplementary Figure 7). The main contributors to the difference in overall benefit–risk scores between maribavir and foscarnet as well as between maribavir and ganciclovir in both ANC cohorts (≥1500/mm^3^ and <1500/mm^3^, respectively) were oral administration (score difference: 0.70 for both cohorts and agent comparisons), lower risk of grade 3–4 neutropenia (score differences: vs foscarnet: 0.46 and 0.88; vs ganciclovir: 0.47 and 0.91), and lower risk of nephrotoxicity (foscarnet comparison only: score difference: 0.36 for both cohorts).

The overall benefit–risk score for ganciclovir was slightly higher than foscarnet in both ANC cohorts, and a more pronounced difference was observed for valganciclovir versus foscarnet (Table 2). The main contributors to the difference between valganciclovir/ganciclovir and foscarnet were nephrotoxicity and, for valganciclovir, oral administration (Supplementary Figure 7).

The predicted choice probabilities for maribavir, valganciclovir, ganciclovir, and foscarnet for the 2 baseline ANC cohorts are shown in Table 2.

## DISCUSSION

The DCE determined how treatment attributes affect physician preferences for preemptive treatments for post-HCT CMV. Physicians prioritized administration and monitoring burden and avoidance of nephrotoxicity and grade 3–4 neutropenia over incremental improvements in viremia clearance, despite established associations between viral load and outcomes [23, 24]. This underscores how treatment-limiting toxicities can erode the clinical value of preemptive anti-CMV therapy.

Physicians were more averse to grade 3–4 neutropenia risk in patients who were already neutropenic at reactivation, consistent with evidence linking severe neutropenia during ganciclovir use to infections and worse outcomes [2, 25]. The impact of nephrotoxicity on physicians’ treatment choices is in line with a previous Delphi panel study in which there was consensus that foscarnet should be avoided in settings of renal dysfunction [26], as well as the known adverse effects of foscarnet on long-term renal function [27].

Physician preference data from the DCE were modeled to determine the trade-offs that physicians are willing to make between week 8 viremia clearance and other attributes. The trade-offs observed between weeks 8 and 16 viremia clearance in this analysis highlight the importance of maintaining viremia clearance through to week 16, suggesting that physicians value durable clearance.

Dysgeusia was included as a DCE attribute, as this was one of the most frequently observed treatment-related adverse events in maribavir-treated patients in phase 3 clinical trials [10, 11]. However, dysgeusia had little impact on physician preference relative to other attributes. This may reflect its subjective nature and lack of standardized grading versus objective measures such as ANC, as well as the fact that dysgeusia is generally self-limiting and not associated with clinically meaningful harm [10, 11, 28, 29]. In addition, dysgeusia commonly occurs in the post-HCT setting, which may further diminish its perceived relevance in treatment decision-making [29, 30].

Physician prioritization of toxicity avoidance may reflect clinicians’ experience managing severe neutropenia in immunocompromised patients, which often requires immediate, intensive intervention. Physician preference for oral administration is likely driven by institutional pressures, including bed availability, infection control considerations, resource utilization, and patient/caregiver burden. Variations in healthcare structures or drug availability patterns may have contributed to differences in physician preferences between countries (Supplementary Results). Further research is warranted to explore why physicians prioritize these attributes when selecting preemptive anti-CMV treatments.

The MCDA primary analysis quantified clinicians' trade-offs between benefit and risk for maribavir versus valganciclovir in post-HCT preemptive CMV treatment, based on the relative importance of treatment attributes derived from the DCE and clinical trial data from the phase 3 AURORA trial. Within the MCDA framework, maribavir was preferred over valganciclovir (ie, it had a higher expected benefit–risk score), largely driven by lower grade 3–4 neutropenia risk, especially in patients with ANC < 1500/mm^3^ at the time of CMV reactivation, despite higher week 8 viremia clearance with valganciclovir versus maribavir in the AURORA trial (77% vs 70%) [11]. Physician preference for maribavir persisted when week 16 viremia clearance was excluded.

When additional clinical data sources were included in the MCDA, maribavir was preferred over valganciclovir, foscarnet, or ganciclovir for the preemptive treatment of post-HCT CMV, which was driven by oral administration and lower risks of grade 3–4 neutropenia (vs valganciclovir/ganciclovir/foscarnet) and nephrotoxicity (vs foscarnet). Although foscarnet is often selected in cytopenic patients [5], the MCDA indicates that neutropenia risk and administration burden still materially influence treatment selection (ie, maribavir vs foscarnet).

The 4-agent comparisons relied on heterogeneous evidence sources with differing populations and endpoints, and some outcomes required assumptions and imputations (Supplementary Table 8). In particular, the SOLSTICE study enrolled patients with refractory/resistant CMV rather than first CMV episodes in the AURORA study, and some safety endpoints, which were not available in SOLSTICE were drawn from other sources [5, 10, 11]. Cidofovir was excluded because it is infrequently used in preemptive treatment as it is limited by renal toxicity [2, 3, 31].

Limitations of the DCE include panel-based recruitment, self-reported preference data, and hypothetical-choice bias [32], although previous research has demonstrated that DCEs can accurately predict real-world choices [33]. We also mitigated these limitations by using clinically reviewed attributes and clear task framing.

Antiviral resistance was not modeled explicitly in the DCE and MCDA, as it was assumed to be indirectly captured by the “viremia clearance at week 8” attribute; patients who develop resistance would be unlikely to achieve virologic clearance by week 8. Cost and access considerations were also excluded from these analyses due to the global nature of the research and the complexity of these considerations across countries, although we acknowledge their critical role in clinical decision-making. No comparison of prior prophylaxis versus no prior prophylaxis was included in the MCDA, as it was not found to have a significant effect on treatment preferences in the DCE. The MCDA model included an a priori assumption that baseline neutropenia would only impact physician preference regarding the risk of neutropenia. However, baseline neutropenia may also influence preferences beyond this modeled interaction.

In conclusion, physicians preferred oral antiviral options with durable viremia clearance and low risks of neutropenia and nephrotoxicity, highlighting the unmet need for effective agents that avoid treatment-limiting toxicity [5, 25, 27, 34]. In the AURORA study, noninferiority of maribavir versus valganciclovir for confirmed CMV viremia clearance at week 8 was not met, and maribavir demonstrated similar CMV viremia clearance during the posttreatment follow-up [11]. Within the framework of the MCDA, maribavir was preferred over valganciclovir, ganciclovir, and foscarnet, mainly due to its favorable benefit–risk profile, that is, oral administration, viremia clearance, and lower neutropenia and nephrotoxicity risk than the other anti-CMV agents.

## Supplementary Material

ofag500_Supplementary_Data

## Supplementary Data

Supplementary materials are available at *Open Forum Infectious Diseases* online. Consisting of data provided by the authors to benefit the reader, the posted materials are not copyedited and are the sole responsibility of the authors, so questions or comments should be addressed to the corresponding author.
