# Supplementary material for: Multi-Criteria Decision Analysis using Physician Preferences from a Global Discrete Choice Experiment to Investigate the Selection of Preemptive Antiviral Treatments for Post-Hematopoietic Cell Transplant Cytomegalovirus Infection
Source: Open Forum Infect Dis. 2026 Sep 1;13(9):ofag500. doi: 10.1093/ofid/ofag500 (PMC13532088; doi:10.1093/ofid/ofag500)
Supplement: Supplementary file 1 [file ofag500_supplementary_data.pdf]

Multi-Criteria Decision Analysis Using Physician Preferences from a Global Discrete Choice Experiment to Investigate the Selection of Preemptive Antiviral Treatments for Post-Hematopoietic Cell Transplant Cytomegalovirus Infection

Miguel-Angel Perales, Jo-Anne H. Young, Tommi Tervonen, Stephane Deparis, Jonathan D. Norton, Cristian Gugiu, Tien Bo, Kimberly Davis, ShunPing Quan, Victoria L. Prince, Adel Abou Ali, Jessica Auciello

ONLINE APPENDIX

Supplemental Methods 3
Supplemental Results 9
Supplemental Table 1: Attributes and levels identified from a targeted literature review 10
Supplemental Table 2: Targeted literature review: PubMed search terms and screening 11
Supplemental Table 3: Data quality variables for the discrete choice experiment 13
Supplemental Table 4. Alpha and beta terms used within the Random Utility Theory framework 14
Supplemental Table 5. Linearity of attributes in the discrete choice experiment using a dummy-coded multinomial logit (MNL) model 16
Supplemental Table 6. Heteroscedastic multinomial logit (HMNL) model for discrete choice experiment data (N=377) 17
Supplemental Table 7. Attribute-level contributions to the benefit-risk score (weight) for maribavir and valganciclovir in the multi-criteria decision analysis for patients with baseline ANC ≥1500/mm^3 (1.5×10^9/L) and <1500/mm^3 (1.5×10^9/L) 18
Supplemental Table 8. Sources for clinical data in multi-criteria decision analysis 19
Supplemental Table 9. Discrete choice experiment participant demographics and characteristics by country 22

Supplemental Table 10. Use of prophylactic agents in HCT recipients in the discrete choice experiment 24
Supplemental Table 11. Minimum acceptable benefit (MAB) of increased probability of viremia clearance at week 8 versus worsening of other attributes (discrete choice experiment) 25
Supplemental Table 12. Overall Benefit-Risk Scores and Predicted Choice Probabilities in Patients with Baseline ANC <1500/mm^3 (1.5×10^9/L) and ≥1500/mm^3 (1.5×10^9/L) in the Multi-Criteria Decision Analysis 26
Supplemental Figure 1. Example choice task from the discrete choice experiment .. 27
Supplemental Figure 2. Participant disposition in the discrete choice experiment...... 28
Supplemental Figure 3. Physician preferences by years of experience, linear-coded interacted multinomial logit (MNL) model (N = 377) 29
Supplemental Figure 4. Physician preferences from the discrete choice experiment by specialization, linear-coded interacted multinomial logit (MNL) model (N = 377) 30
Supplemental Figure 5. Physician preferences from the discrete choice experiment by country, linear-coded interacted multinomial logit (MNL) model (N = 377) 31
Supplemental Figure 6. Probabilistic sensitivity analysis of attribute-level contributions to the benefit-risk score for maribavir and valganciclovir in patients with baseline absolute neutrophil count a) ≥1500/mm^3 (1.5×10^9/L) and b) <1500/mm^3 (1.5×10^9/L) in the multi-criteria decision analysis........ 33
Supplemental Figure 7. Attribute-level contributions to the benefit-risk score for valganciclovir, maribavir, ganciclovir, and foscarnet in the multi-criteria decision analysis for patients with baseline absolute neutrophil count a) ≥1500/mm^3 (1.5×10^9/L) and b) <1500/mm^3 (1.5×10^9/L) 34
References........ 35

Supplementary Methods

Discrete Choice Experiment

Cognitive pilot interviews

The DCE was pre-tested in cognitive pilot interviews (July 19–August 23, 2024) with 28 participants to assess survey clarity and identify any issues with the user interface. For the cognitive pilot interviews, participants, who were willing to be audio-recorded, were provided with a link to the survey and access details for a video conferencing interview. During the 60-minute web-assisted interview, participants were asked to complete the survey and to provide feedback on any unclear content or challenges with the user interface. The cognitive pilot interviews were conducted in the local language by native-speaker interviewers. These interviews were conducted in an iterative manner where each iteration or wave contained a subset of participants. Any problems identified in a wave of interviews were analyzed and corrected prior to proceeding to the following round. The first round was conducted with half of the pilot-phase sample, and after updates based on the first-round results, a second round was conducted with the remaining participants. English-speaking countries were prioritized in the pilot interviews for the first round. A quantitative pilot/soft launch of the survey was also conducted with a subset of participants to ensure that the DCE captured trade-offs appropriately.

Targeted literature review

A targeted literature review (TLR) was conducted to understand the recent literature on patient and/or physician preferences and effectiveness and/or safety of non-prophylactic treatments for post-hematopoietic cell transplant (HCT) cytomegalovirus (CMV). The objective of the TLR was to develop a draft effects table describing key clinical data for first-line preemptive treatment of post-HCT CMV. The TLR focused on published peer-reviewed articles identified on PubMed via pre-defined search strategies (**Supplemental Table 1**). Additional hand-picked articles (e.g., from reference lists) were included. Articles were single-screened by 1 investigator from Kielo (Zug, Switzerland), and potential inclusions reviewed by another investigator. Eligibility criteria for the clinical trial and real-world studies were updated during the review to exclude studies without useful data for the discrete choice experiment (DCE) and multi-criteria

decision analysis (MCDA; i.e., those with non-standard dosing or without comparative efficacy or safety data).

The targeted literature review for treatment preferences in cytomegalovirus included preference studies in CMV or HCT, qualitative interview studies with patients or clinicians on treatment perspectives of CMV or HCT, and health state utility studies in CMV or HCT. Clinical trials and non-English articles were excluded. A total of 53 records were identified on PubMed, of which 51 were excluded after screening the abstracts, and the remaining 2 publications were excluded following screening of the full text. Therefore, no studies were included in the review.

The targeted literature review on clinical trials and real-world studies on the relevant treatments included clinical trials on treatment efficacy or safety or observational studies on treatment effectiveness or safety for treatments of interest in post-HCT CMV, and systematic reviews. The following types of articles were excluded: non-English articles, opinion articles, non-systematic reviews, and studies evaluating prophylactic approaches, involving a non-adult population or non-standard dosing, or with no comparative or safety data. A total of 253 records were identified on PubMed, and 3 articles were identified manually. Following screening of the abstracts, 237 articles were excluded. Full text was screened for the remaining 19 articles and 9 were excluded. Therefore, 10 studies were included in the review, including 1 systematic literature review [1], 2 randomized clinical trials [2, 3], 4 open label clinical trials / prospective studies [4-7], and 4 retrospective studies [4, 8-10] (1 study had both retrospective and prospective cohorts [4]).

Randomization and design of forced-choice tasks

Each physician completed 15 experimental choice tasks chosen from 2 blocks of 15 choice tasks. In addition, 2 non-experimental tasks were included in the DCE to test the internal validity of participants' responses. The first non-experimental choice task (second-to-last choice task) repeated the first-choice task and was used to test the stability of participants' preferences [11]. The second non-experimental task (last choice task) was a dominated choice task where 1 alternative was clearly better because it was superior (or equal) on every attribute; it was used to assess participants' attention and

understanding of the tasks [12]. Attribute level combinations in the choice tasks were chosen using a d-efficient experimental design developed using Ngene software [13].

Attribute order was randomized, with attribute randomization implemented in 3 attribute groups (i.e., benefit, risk and administration). The order of risk attributes and the 3 groups were randomized. All randomization was between participants, i.e., each participant saw attributes in the same order in all choice tasks. The order of the experimentally designed choice tasks was randomized between participants, and participants were randomly assigned to 1 of the 2 blocks of choice tasks. The non-experimental choice tasks (repeated and dominated choices) were always presented as the 2 last ones.

Data quality check

Data quality variables were constructed from DCE responses to assess the consistency and attention of participants. These included dominated choice to check if participants chose the dominant alternative (i.e., superior option on every attribute), or repeated choice to check if participants chose the same answer in repeated choice questions. The data were also checked for straight lining in which participants selected the corresponding alternative (e.g. All A or all B) in all choice tasks, and for attribute dominance in which participants selected the option with the “better” value in that given attribute for all choice tasks, and thus may have been focusing on a single attribute and not trading off between attributes. Participants were not excluded from the analysis based on these data quality variables, except for straight lining. Completion times for the survey were also analyzed. Data quality variables are summarized in Supplemental Table 3**.**

Statistical Methods

The formula used within the Random Utility Theory framework was:

$$u(x) = \alpha_{left} + \beta_{w8} * x_{w8} + \beta_{w16} * x_{w16} + \beta_{neu} * x_{neu} + \beta_{thr} * x_{thr} + \beta_{dys} * x_{dys} + \beta_{tox} * x_{tox} + \beta_{adm.IV} * x_{adm.IV} + \beta_{adm.O} * x_{adm.O} + i_{pro} * x_{pro} * \beta_{neu} + i_{neu} * x_{neu} * \beta_{neu} + \varepsilon$$

The alpha and beta terms are described in Supplemental Table 4, x_a are the measurements of *x* on *a*, either risk, or for the administration modes, 0/1. The EV-1 distributed error term is ε.

The linearity of preferences was tested for all attributes except administration and monitoring by fitting a linear model to the coefficients of the dummy-coded model and assessing R^2. All attributes were found to be linear at $R^2 > 0.8$ (the lowest R^2 was 0.88 for the risk of dysgeusia; Supplemental Table 5), and linear coding was used in all further analyses for all attributes apart from administration and monitoring.

A heteroscedastic multinomial logit model (HMNL) was then estimated to determine whether DCE data from different countries could be pooled together; the US was chosen as the reference category as it had the most participants. HMNL results indicate significant scale heterogeneity only between Australia versus the US ($P = 0.025$; Supplemental Table 6). Given the low number of participants in Australia, the scale differences were deemed acceptable and data from all countries were pooled without adjusting for scale differences in further analyses.

The relative attribute importance (RAI) score was calculated as the difference in utilities between the best or most preferred level of an attribute and the worst or least preferred level of the same attribute using the following formula:

$$\mathrm{RAI}_k = 100 \times \frac{\hat{\beta}_k^{\max} - \hat{\beta}_k^{\min}}{\sum_{k\in[1;K]}\left(\hat{\beta}_k^{\max} - \hat{\beta}_k^{\min}\right)}$$

Where k is the attribute and $(\hat{\beta}_k^{\max} - \hat{\beta}_k^{\min})$ corresponds to the largest change in utility obtained with moving from the worst (least desirable) to best (most desirable) level of the kth attribute. If the worst level also happens to be the reference category for the dummy coding of the effects, then $\hat{\beta}_k^{\min} = 0\hat{\beta}_{\mathrm{k}}^{\min} = 0$.

Multi-criteria decision analysis

The multi-criteria decision analysis (MCDA) combined treatment performance data on each attribute with their relative importance weights, which were obtained from the discrete choice experiment (DCE). An additive value model was used to compare

treatments based on their benefit-risk scores (V_i), calculated as follows for each treatment:

$$V_i = \sum_{j=1}^{J} \beta_j \mu_{i,j}$$

Here, $\boldsymbol{\mu_{i,j}}$ is the performance of treatment i for attribute j (based on the clinical trial data), and J is the total number of attributes considered (i.e., benefits and risks). The weights β_j for each attribute j were obtained from the corresponding linear-coded coefficients of the DCE. The treatment i with the highest benefit-risk score was considered the best.

In addition to using the additive value model, treatments were also compared based on predicted choice probabilities using the logit formula based on random utility theory [14-16]:

$$P_i = \frac{\exp\,(V_i)}{\Sigma_{k \epsilon Y} \exp\,(V_k)'}$$

where Y is the set of treatments considered. The predicted choice probabilities express how likely a treatment i is to be chosen by any single physician. Random utility theory assumes that a decision maker will choose the alternative with the highest utility, in this case based on the weights and observed data. The formula includes a random error term (Σ) with an extreme value type 1 distribution making it functionally equivalent to logistic regression.

The impact of the uncertainty of clinical effect estimates in the benefit-risk scores was assessed for the primary MCDA analysis (maribavir vs valganciclovir) using bootstrapping on the patient-level data from AURORA to generate 95% confidence intervals (CIs) for the benefit-risk scores. The impact of uncertainty of preference estimates was assessed using Krinsky-Robb bootstrapping [17] and was reflected in the 95% CIs for predicted choice probabilities.

A probabilistic sensitivity analysis was also undertaken in which clinical and preference uncertainty were both taken into account when calculating the benefit-risk scores and CIs. Clinical uncertainty was simulated by bootstrapping clinical trials endpoint data (*N* times). Preference uncertainty was simulated by drawing *N* sample

weights from a multivariate normal (MVN) distribution, using DCE coefficient means and their variance-covariance matrix. The bootstrapped clinical matrices were multiplied by the MVN-sampled weight matrices to generate a joint distribution of benefit-risk scores for both patient cohorts: those with or without prior neutropenia.

To properly account for joint uncertainty for predicted choice probabilities, the logit formula was applied at the simulation level. For each of the iterations, the simulated clinical performance data and the simulated preference weights were multiplied to create a total benefit-risk score, which was then transformed into a probability. The distributions of these probabilities provided final estimates and 95% CIs.

Supplemental Results

Discrete Choice Experiment

Physician preference by country

Physician preference heterogeneity by country is presented in Supplemental Figure 5. As compared with US participants, Australian participants valued week 16 viremia clearance more (*P* = 0.005), and the impact of the neutropenic patient profile was higher (*P* = 0.03). French participants valued oral administration less than those from the United States (*P* = 0.03), and the impact of the neutropenic patient profile was higher (*P* = 0.006). German participants valued lower risks of dysgeusia (*P* = 0.02) and nephrotoxicity (*P* = 0.03) more, and the impact of previous prophylaxis in the patient profile was lower (*P* = 0.04). Italian participants valued a lower risk of neutropenia (*P* = 0.03) and oral administration (*P* = 0.02) less. The impact of the neutropenic patient profile was higher (*P* = 0.03). No significant differences (versus participants from the US) were detected in the preferences of participants from Canada, Spain and the UK.

Supplemental Table 1. Attributes and levels identified from a targeted literature review

Attribute	Definition	Levels
Viremia clearance at week 8	Chance of achieving CMV viremia clearance (DNAemia under the LLOQ) at the end of 8 weeks of treatment	60%; 70%; and 80%
Viremia clearance at week 16	Chance of maintaining CMV viremia clearance (DNAemia under the LLOQ) for another 8 weeks off treatment	40%; 50%; and 60%
Risk of grade 3–4 neutropenia	Risk of developing grade 3–4* neutropenia (ANC <1,000/mm^3 [<1.0×10^9/L])	15%; 35%; and 50%
Risk of thrombocytopenia	Risk of developing thrombocytopenia (platelet count <150,000/mm^3 [<150×10^9/L])	5%; 15%; and 30%
Risk of dysgeusia	Risk of developing dysgeusia (taste abnormality)	5%; 25%; and 40%
Risk of nephrotoxicity	Risk of abnormal renal function (abnormally high concentration of creatinine in the blood), renal impairment, or grade 2–4† acute kidney injury (creatinine 2 to 3 times above baseline or greater)	5%; 25%; and 40%
Administration and monitoring	Treatment administration and monitoring requirements	Oral tablets not requiring hospitalization for administration;<br>IV administration on an outpatient basis;<br>IV administration requiring hospitalization and monitoring

*Grading as per Common Terminology Criteria for Adverse Events Version 5. †Grading as per Common Terminology Criteria for Adverse Events Version 4.
ANC, absolute neutrophil count; CMV, cytomegalovirus; DCE, Discrete Choice Experiment; IV, intravenous; LLOQ, lower limit of quantification.

Supplemental Table 2. Targeted literature review: PubMed search terms and screening

A. Treatment preferences in cytomegalovirus

#	Description	Search Terms	Number of Hits	Cumulative hits
**1**	Population	Patient*[tiab] OR clinician*[tiab] OR physician*[tiab] OR doctor*[tiab] OR oncologist*[tiab] OR surgeon*[tiab] OR virologist*[tiab]	8,729,348	8,729,348
**2**	Indication	Cytomegalovir*[tiab] OR cmv[tiab] OR hematopoietic*[tiab] OR hsct*[tiab] OR hct[tiab]	190,314	82,641
**3**	Study type	Discrete-choice*[tiab] OR discrete choice*[tiab] OR choice experiment*[tiab] OR conjoint*[tiab] OR best worst*[tiab] OR best-worst*[tiab] OR threshold*[tiab] OR interview*[tiab] OR focus group*[tiab] OR survey*[tiab] OR swing weight*[tiab] OR mcda[tiab] OR multi-criteria*[tiab] OR multi criteria*[tiab] OR utilit*[tiab] OR health state*[tiab]	1,865,983	3,377
**4**	Outcome	preference*[tiab]	197,507	56
**5**	Other	full text[sb]	-	**53**

B. Clinical trials and real-world studies on the relevant treatments

#	Description	Search Terms	Number of Hits	Cumulative hits
**1**	Population	Hematopoietic*[tiab] OR hsct*[tiab] OR bone marrow*[tiab] OR hct[tiab]	351,155	351,155
**2**	Indication	Cytomegalovir*[tiab] OR cmv[tiab]	57,392	5,065
**3**	Intervention	valganciclovir[tiab] OR ganciclovir[tiab] OR foscarnet[tiab] OR maribavir[tiab] OR cidofovir[tiab] OR acyclovir[tiab] OR letermovir[tiab]	18,936	880
**4**	Study type	randomized controlled trial[Publication Type] OR randomized[Title/Abstract] OR controlled[Title/Abstract] OR trial[Title/Abstract] OR observational[tiab] OR retrospective[tiab] OR case control[tiab] OR prospective[tiab] OR cohort[tiab] OR cross-sectional[tiab] OR cross sectional[tiab] OR longitudinal[tiab]	4,356,681	283
**5**	Other	full text[sb]	-	**253**

Supplemental Table 3. Data quality variables for the discrete choice experiment

Measure n (%)	Overall n = 377	Australia n = 24	Canada n = 42	France n = 48	Germany n = 46	Italy n = 51	Spain n = 51	United Kingdom n = 51	United States n = 64	p-value*
**Dominated choice correct**	320 (85)	21 (88)	38 (90)	43 (90)	43 (93)	43 (84)	40 (78)	37 (73)	55 (86)	0.128
**Repeated choice same**	265 (70)	18 (75)	32 (76)	35 (73)	35 (76)	34 (67)	30 (59)	36 (71)	45 (70)	0.613
**Straight-lining**										0.818
All A	2 (0.5)	0 (0)	0 (0)	0 (0)	1 (2.2)	0 (0)	0 (0)	0 (0)	1 (1.6)	
All B	0 (0)	0 (0)	0 (0)	0 (0)	0 (0)	0 (0)	0 (0)	0 (0)	0 (0)	
Neither	375 (99)	24 (100)	42 (100)	48 (100)	45 (98)	51 (100)	51 (100)	51 (100)	63 (98)	
**Time to complete survey**										0.155
<5 min	63 (17)	1 (4.2)	6 (14)	14 (29)	7 (15)	11 (22)	7 (14)	9 (18)	8 (13)	
5–9.59 min	112 (30)	10 (42)	10 (24)	6 (13)	10 (22)	18 (35)	20 (39)	15 (29)	23 (36)	
10–20 min	120 (32)	9 (38)	14 (33)	14 (29)	17 (37)	17 (33)	14 (27)	15 (29)	20 (31)	
>20 min	82 (22)	4 (17)	12 (29)	14 (29)	12 (26)	5 (9.8)	10 (20)	12 (24)	13 (20)	
**Attribute dominance**										
Viremia clearance at week 8	3 (0.8)	0 (0)	0 (0)	0 (0)	0 (0)	1 (2.0)	0 (0)	1 (2.0)	1 (1.6)	
Viremia clearance at week 16	2 (0.5)	2 (8.3)	0 (0)	0 (0)	0 (0)	0 (0)	0 (0)	0 (0)	0 (0)	
Risk of G3–4 neutropenia	3 (0.8)	0 (0)	2 (4.8)	0 (0)	0 (0)	0 (0)	0 (0)	0 (0)	1 (1.6)	
Risk of thrombocytopenia	0 (0)	0 (0)	0 (0)	0 (0)	0 (0)	0 (0)	0 (0)	0 (0)	0 (0)	
Risk of dysgeusia	0 (0)	0 (0)	0 (0)	0 (0)	0 (0)	0 (0)	0 (0)	0 (0)	0 (0)	
Risk of nephrotoxicity	7 (1.9)	1 (4.2)	2 (4.8)	1 (2.1)	1 (2.2)	0 (0)	0 (0)	1 (2.0)	1 (1.6)	
Administration and monitoring	10 (2.7)	1 (4.2)	2 (4.8)	1 (2.1)	1 (2.2)	0 (0)	0 (0)	4 (7.8)	1 (1.6)	

*Fisher's Exact Test for Count Data with simulated p-value (based on 2000 replicates); Pearson's Chi-squared test; Fisher's exact test
G, grade

Supplemental Table 4. Alpha and beta terms used within the Random Utility Theory framework

**Viremia clearance at week 8**	60%	Reference
	70%	$\beta_{w8.70}$
	80%	$\beta_{w8.80}$
	Linear – per 1%	β_{w8}
**Viremia clearance at week 16**	40%	Reference
	50%	$\beta_{w16.50}$
	60%	$\beta_{w16.60}$
	Linear – per 1%	β_{w16}
**Risk of G3-4 neutropenia**	50%	Reference
	35%	$\beta_{neu.35}$
	15%	$\beta_{neu.15}$
	Linear – per 1%	β_{neu}
**Risk of thrombocytopenia**	30%	Reference
	15%	$\beta_{thr.15}$
	5%	$\beta_{thr.5}$
	Linear – per 1%	β_{thr}
**Risk of dysgeusia**	40%	Reference
	25%	$\beta_{dys.25}$
	5%	$\beta_{dys.5}$
	Linear – per 1%	β_{dys}
**Risk of nephrotoxicity**	40%	Reference
	25%	$\beta_{tox.25}$
	5%	$\beta_{tox.5}$
	Linear – per 1%	β_{tox}
**Administration and monitoring**	Intravenous administration requiring hospitalization and monitoring	Reference
	Intravenous administration which can be on an outpatient basis	$\beta_{adm.IV}$

	Oral tablets that do not require hospitalization for administration	$\beta_{adm.O}$
**Constants**		**Parameters**
**Left alternative**		α_{left}
**Effect of patient profile (interaction terms with Risk of Neutropenia)**		**Parameters**
**CMV prophylaxis**	Patient did not receive CMV prophylaxis	Reference
	Patient received CMV prophylaxis	i_{pro}
**Neutropenia**	Patient does not have neutropenia	Reference
	Patient has neutropenia	i_{neu}

Supplemental Table 5. Linearity of attributes in the discrete choice experiment using a dummy-coded multinomial logit (MNL) model

Attribute	R^2
Viremia clearance at week 8	0.99
Viremia clearance at week 16	0.97
Risk of grade 3–4 neutropenia	0.96
Risk of thrombocytopenia	0.91
Risk of dysgeusia	0.88
Risk of nephrotoxicity	0.98

Supplemental Table 6. Heteroscedastic multinomial logit (HMNL) model for discrete choice experiment data (N=377)

Parameter	Estimate	SE	CI	*P*-value
Intercept (left alternative)	0.082	0.031	[0.022 to 0.142]	0.007
Viremia clearance at week 8 (continuous)	0.021	0.003	[0.016 to 0.027]	0.000
Viremia clearance at week 16 (continuous)	0.014	0.002	[0.009 to 0.019]	0.000
Risk of G3-4 neutropenia (continuous)	-0.014	0.002	[-0.019 to -0.01]	0.000
Risk of thrombocytopenia (continuous)	-0.006	0.002	[-0.01 to -0.003]	0.001
Risk of dysgeusia (continuous)	-0.005	0.001	[-0.007 to -0.002]	0.000
Risk of nephrotoxicity (continuous)	-0.015	0.002	[-0.018 to -0.011]	0.000
Admin: IV	0.222	0.046	[0.131 to 0.313]	0.000
Admin: Oral	0.710	0.070	[0.572 to 0.847]	0.000
Prophylaxis * neutropenia (per 10%)	-0.005	0.025	[-0.054 to 0.044]	0.841
Has neutropenia * neutropenia (per 10%)	0.130	0.027	[0.078 to 0.182]	0.000
Scale factor: Australia	0.189	0.084	[0.024 to 0.355]	0.025
Scale factor: Canada	0.094	0.068	[-0.04 to 0.227]	0.170
Scale factor: France	-0.046	0.063	[-0.17 to 0.079]	0.472
Scale factor: Germany	0.022	0.065	[-0.106 to 0.151]	0.731
Scale factor: Italy	-0.106	0.061	[-0.226 to 0.014]	0.084
Scale factor: Spain	-0.041	0.062	[-0.162 to 0.08]	0.510
Scale factor: UK	-0.039	0.062	[-0.16 to 0.082]	0.529

CI, confidence interval; IV, intravenous, SE, standard error.

Supplemental Table 7. Attribute-level contributions to the benefit-risk score (weight) for maribavir and valganciclovir in the multi-criteria decision analysis for patients with baseline ANC ≥1500/mm^3 (1.5×10^9/L) and <1500/mm^3 (1.5×10^9/L) based on the homoscedastic model

Attribute	Weight (ANC ≥1500/mm^3)	Weight (ANC <1500/mm^3)
Viremia clearance at 8 weeks (end of therapy)	0.0217	0.0217
Viremia clearance at 16 weeks (8 weeks off treatment)	0.0131	0.0131
Grade 3–4 neutropenia (ANC <1000/mm^3 [<1.0×10^9/L])	−0.0140	−0.0268
Thrombocytopenia	−0.0064	−0.0064
Dysgeusia	−0.0047	−0.0047
Nephrotoxicity	−0.0143	−0.0143
Administration	0.6967	0.6967

ANC, absolute neutrophil count.

Supplemental Table 8. Sources for clinical data in the multi-criteria decision analysis

**Analyses comparing maribavir and valganciclovir**			
**Attribute**	**Description**	**Maribavir[a]**	**Valganciclovir[a]**
Viremia clearance at 8 weeks (end of therapy)	Percentage of patients with viremia clearance at week 8	190/273 (69.6%)	212/274 (77.4%)
Viremia clearance at 16 weeks (8 weeks off treatment)	Percentage of patients with viremia clearance at week 16	144/273 (52.7%)	133/274 (48.5%)
Grade 3–4 neutropenia	Grade 3–4 neutropenia (TEAE) (ANC <1000/mm^3 [<1.0×10^9/L])	44/273 (16.1%)	137/274 (50.0%)
Thrombocytopenia	Any grade thrombocytopenia (TEAE)	31/273 (11.4%)	63/274 (23.0%)
Dysgeusia	Abnormal taste, max. grade is 2 (TEAE)	47/273 (17.2%)	16/274 (5.8%)
Nephrotoxicity	Renal impairment / abnormal renal function / grade 2–4 acute kidney injury (TEAE)	25/273 (9.2%)	15/274 (5.5%)
Administration	Mode of administration	Oral	Oral

Analyses comparing maribavir, valganciclovir, ganciclovir, and foscarnet				
**Attribute**	**Maribavir**	**Valganciclovir**	**Ganciclovir[b]**	**Foscarnet**
Viremia clearance at 8 weeks (end of therapy) [3, 7][c]	190/273 (69.6%)[a]	212/274 (77.4%)[a]	212/274 (77.4%)[a]	Assume same rate as valganciclovir
Viremia clearance at 16 weeks (8 weeks off treatment) [3, 7][c]	144/273 (52.7%)[a]	133/274 (48.5%)[a]	133/274 (48.5%)[a]	Assume same rate as valganciclovir
Grade 3–4 neutropenia (ANC <1000/mm^3 [<1.0×10^9/L]) [3, 10][d]	44/273 (16.1%)[a]	137/274 (50.0%)[a]	137/274 (50.0%)[a]	23/47 (48.9%)
Thrombocytopenia [18][d]	6/92 (6.5%)	5/20 (25.0%)	5/20 (25.0%)	1/18 (5.6%)
Dysgeusia [18][d]	25/92 (27.2%)	1/20 (5.0%)	1/20 (5.0%)	0/18
Nephrotoxicity [3, 10][d]	25/273 (9.2%)[a]	15/274 (5.5%)[a]	15/274 (5.5%)[a]	16/47 (34.0%)
Administration [19-22]	Oral	Oral	IV	IV requiring hospitalization and monitoring

[a]Data sourced from the AURORA study [3].

[b]Assumed that the efficacy and safety data for ganciclovir is same as valganciclovir based on results of VICTOR study [23]:

- Viremia clearance met non-inferiority criteria at Day 21: valganciclovir: 45.1% vs ganciclovir: 48.4% (95% CI for difference: −14.0 to +8.0%); viremia clearance at Day 49: 67.1% vs 70.1%, respectively (p = not significant)
- No major differences reported in frequencies of adverse events between treatment groups.

[c]Assumed that viremia clearance at 8 weeks (end of treatment) and after a further 8 weeks of follow-up would be the same for foscarnet, ganciclovir, and valganciclovir based on the following data:

- Foscarnet versus ganciclovir: CMV detectable at end of: 14-day induction period: 29% vs 34% (p=0.4); 14-day maintenance period: 10% vs 13% (p=0.5); retreatment required within 180 days after HCT: 43% vs 28% (p=0.6) [7]
- SOLSTICE study: valganciclovir/ganciclovir vs foscarnet: week 8 viremia clearance: 26.8% vs 19.1% [24]
- VICTOR study: see footnote b [23].

[d]For dysgeusia and thrombocytopenia, data from SOLSTICE study were used, as all 4 treatments were evaluated in this study [24]; as grade 3–4 neutropenia and nephrotoxicity data were not available in SOLSTICE for individual agents, data from AURORA were used for maribavir, valganciclovir and ganciclovir and from a retrospective study evaluating foscarnet and valganciclovir for foscarnet [3, 10].

[e]Grading as per Common Terminology Criteria for Adverse Events Version 5.

[f]Grading as per Common Terminology Criteria for Adverse Events Version 4.

Abbreviations: ANC, absolute neutrophil count; CI, confidence interval; CMV, cytomegalovirus; IV, intravenous; LLOQ, lower limit of quantification; MCDA, multi-criteria decision analysis; TEAE, treatment-emergent adverse event.

Supplemental Table 9. Discrete choice experiment participant demographics and characteristics by country

Characteristic	Australia n = 24	Canada n = 42	France n = 48	Germany n = 46	Italy n = 51	Spain n = 51	UK n = 51	US n = 64
**Age, years**								
Mean (SD)	43.6 (9.2)	50.6 (8.7)	46.7 (6.6)	49.0 (6.7)	46.9 (10.0)	42.9 (8.3)	46.1 (7.1)	46.5 (9.8)
Median (range)	41 (31, 60)	51 (34, 68)	47 (33, 64)	49 (32, 63)	46 (31, 70)	42 (28, 59)	45 (31, 58)	48 (30, 76)
**Sex, n (%)**								
Male	16 (66.7)	33 (78.6)	35 (72.9)	39 (84.8)	36 (70.6)	31 (60.8)	36 (70.6)	46 (71.9)
Female	8 (33.3)	9 (21.4)	13 (27.1)	7 (15.2)	15 (29.4)	20 (39.2)	15 (29.4)	18 (28.1)
**Years experience treating patients with CMV infections post-HCT, n (%)**								
>2 to ≤3	1 (4.2)	0 (0)	2 (4.2)	0 (0)	2 (3.9)	0 (0)	2 (3.9)	8 (12.5)
>3 to ≤4	2 (8.3)	1 (2.4)	2 (4.2)	0 (0)	3 (5.9)	5 (9.8)	4 (7.8)	7 (10.9)
≥4	21 (87.5)	41 (97.6)	44 (91.7)	46 (100)	46 (90.2)	46 (90.2)	45 (88.2)	49 (76.6)
**Years managing HCT patients, n (%)**								
0–5	7 (29.2)	3 (7.1)	1 (2.1)	0 (0)	5 (9.8)	6 (11.8)	8 (15.7)	11 (17.2)
6–10	9 (37.5)	9 (21.4)	10 (20.8)	8 (17.4)	12 (23.5)	17 (33.3)	15 (29.4)	14 (21.9)
11–20	6 (25.0)	17 (40.5)	26 (54.2)	32 (69.6)	19 (37.3)	17 (33.3)	23 (45.1)	25 (39.1)
>20	2 (8.3)	13 (31.0)	11 (22.9)	6 (13.0)	15 (29.4)	11 (21.6)	5 (9.8)	14 (21.9)
**Number of HCT patients seen on average per month, n (%)**								
<2	5 (20.8)	3 (7.1)	1 (2.1)	0 (0)	4 (7.8)	4 (7.8)	7 (13.7)	10 (15.6)
3–5	3 (12.5)	17 (40.5)	15 (31.3)	11 (23.9)	8 (15.7)	6 (11.8)	6 (11.8)	12 (18.8)
6–10	9 (37.5)	8 (19.0)	14 (29.2)	16 (34.8)	11 (21.6)	19 (37.3)	14 (27.5)	16 (25.0)
>10	7 (29.2)	14 (33.3)	18 (37.5)	19 (41.3)	28 (54.9)	22 (43.1)	24 (47.1)	26 (40.6)

**Are there guidelines on post-HCT CMV management where you work, n (%)**								
Yes, the guidelines dictate which treatments need to be used	15 (62.5)	17 (40.5)	21 (43.8)	12 (26.1)	15 (29.4)	21 (41.2)	27 (52.9)	26 (40.6)
Yes, but I can choose any treatment for CMV	9 (37.5)	22 (52.4)	22 (45.8)	33 (71.7)	30 (58.8)	27 (52.9)	22 (43.1)	27 (42.2)
No	0 (0)	3 (7.1)	5 (10.4)	1 (2.2)	6 (11.8)	3 (5.9)	2 (3.9)	11 (17.2)
**Specialty, n (%)***								
Hematology	14 (58.3)	27 (64.3)	28 (58.3)	33 (71.7)	35 (68.6)	35 (68.6)	33 (64.7)	23 (35.9)
Oncology	3 (12.5)	25 (59.5)	20 (41.7)	27 (58.7)	16 (31.4)	22 (43.1)	19 (37.3)	24 (37.5)
Infectious disease specialist	8 (33.3)	10 (23.8)	14 (29.2)	14 (30.4)	12 (23.5)	18 (35.3)	10 (19.6)	32 (50.0)
HCT specialist	9 (37.5)	19 (45.2)	16 (33.3)	22 (47.8)	20 (39.2)	21 (41.2)	26 (51.0)	16 (25.0)

*Participants were permitted to select more than 1 specialty.
CMV, cytomegalovirus; HCT, hematopoietic cell transplant.

Supplemental Table 10. Use of prophylactic agents in HCT recipients in the discrete choice experiment*

Characteristic	Australia n = 24	Canada n = 42	France n = 48	Germany n = 46	Italy n = 51	Spain n = 51	UK n = 51	US n = 64	Overall N = 377
Letermovir	9 (37.5)	21 (50.0)	27 (56.3)	25 (54.3)	36 (70.6)	24 (47.1)	24 (47.1)	47 (73.4)	213 (56.5)
Ganciclovir	10 (41.7)	30 (71.4)	23 (47.9)	26 (56.5)	35 (68.6)	33 (64.7)	33 (64.7)	30 (46.9)	220 (58.4)
Valganciclovir	14 (58.3)	25 (59.5)	28 (58.3)	30 (65.2)	33 (64.7)	37 (72.5)	36 (70.6)	44 (68.8)	247 (65.5)
Foscarnet	3 (12.5)	16 (38.1)	15 (31.3)	14 (30.4)	21 (41.2)	23 (45.1)	22 (43.1)	12 (18.8)	126 (33.4)
Acyclovir	7 (29.2)	14 (33.3)	16 (33.3)	19 (41.3)	22 (43.1)	25 (49.0)	30 (58.8)	18 (28.1)	151 (40.1)
Cidofovir	0 (0)	6 (14.3)	8 (16.7)	12 (26.1)	12 (23.5)	9 (17.6)	15 (29.4)	9 (14.1)	71 (18.8)
Other	1 (4.2)	3 (7.1)	0 (0)	0 (0)	0 (0)	0 (0)	0 (0)	1 (1.6)	5 (1.3)
I do not use CMV prophylactic agents in patients who received HCT	2 (8.3)	1 (2.4)	2 (4.2)	1 (2.2)	0 (0)	0 (0)	0 (0)	1 (1.6)	7 (1.9)

*Participants were permitted to select more than 1 agent.
CMV, cytomegalovirus; HCT, hematopoietic cell transplant.

Supplemental Table 11. Minimum acceptable benefit (MAB) of increased probability of viremia clearance at week 8 versus worsening of other attributes (discrete choice experiment)

Measure	MAB	95% CI
Viremia clearance at week 16 (for 10% decrease)	6.0	[4–8.6]
Risk of G3–4 neutropenia (for 10% increase)	6.5	[4.4–9.1]
Risk of thrombocytopenia (for 10% increase)	2.9	[1.3–4.9]
Risk of dysgeusia (for 10% increase)	2.2	[1.1–3.4]
Risk of nephrotoxicity (for 10% increase)	6.6	[5–8.7]
Admin: IV, hosp. + monitoring instead of IV	9.6	[5.7–14.2]
Admin: IV, hosp. + monitoring instead of Oral	32.1	[26–40.7]

CI, confidence interval; G, grade; IV, intravenous.

Table reproduced from Perales MA, Tervonen T, Deparis S, et al. Insights from a Discrete Choice Experiment: How Benefits and Risks Affect Physician Preferences for Pre-emptive CMV Treatment in Hematopoietic Cell Transplant Recipients [Abstract]. 51st EBMT Annual Meeting, March 30–April 2, 2025, Florence, Italy

Supplemental Table 12. Overall Benefit-Risk Scores and Predicted Choice Probabilities in Patients with Baseline ANC <1500/mm^3 (1.5×10^9/L) and ≥1500/mm^3 (1.5×10^9/L) in the Multi-Criteria Decision Analysis. Bootstrapping was employed for both types of uncertainty in this sensitivity analysis.

**Maribavir versus valganciclovir**		
	**Maribavir**	**Valganciclovir**
**Baseline ANC ≥1500/mm^3 (1.5×10^9/L)**		
**Benefit-risk score (95% CI)**	2.390 (1.959–2.824)	2.060 (1.556–2.563)
**Benefit-risk score difference vs valganciclovir (95% CI)**	0.330 (0.025–0.643)	
**Predicted Choice Probabilities* (95% CI)**	0.581 (0.506–0.655)	0.419 (0.345–0.494)
**Baseline ANC <1500/mm^3 (1.5×10^9/L)**		
**Benefit-risk score (95% CI)**	2.184 (1.751–2.626)	1.419 (0.925–1.909)
**Benefit-risk score difference vs valganciclovir (95% CI)**	0.765 (0.430–1.113)	
**Predicted Choice Probabilities* (95% CI)**	0.681 (0.606–0.753)	0.319 (0.247–0.394)

*Predicted Choice Probability expresses how likely, on average, a treatment is to be chosen from a set of options considered by a physician

Abbreviations: ANC, absolute neutrophil count; CI, confidence interval.

Supplemental Figure 1. Example choice task from the discrete choice experiment

Which preemptive treatment would you choose for a patient who:
Received CMV prophylaxis for 100 days, and CMV reactivation occurred after that, and
Has neutropenia?

	Feature	Treatment A	Treatment B
BENEFITS	**CMV viremia clearance**	Clear at week 8: 60% (60 out of 100)<br>Still clear at week 16: 50% (50 out of 100)	Clear at week 8: 70% (70 out of 100)<br>Still clear at week 16: 40% (40 out of 100)
RISKS	**Risk of grade 3–4 neutropenia**	35% (35 of 100)	50% (50 of 100)
	**Risk of thrombocytopenia**	15% (15 of 100)	30% (30 of 100)
	**Risk of dysgeusia**	25% (25 of 100)	40% (40 of 100)
	**Risk of nephrotoxicity**	40% (40 of 100)	25% (25 of 100)
ADMIN	**Administration and monitoring**	Oral	IV<br>+ hospitalization<br>+ monitoring
	CHOICE	◌	◌

CMV, cytomegalovirus, IV, intravenous

Supplemental Figure 2. Participant disposition in the discrete choice experiment

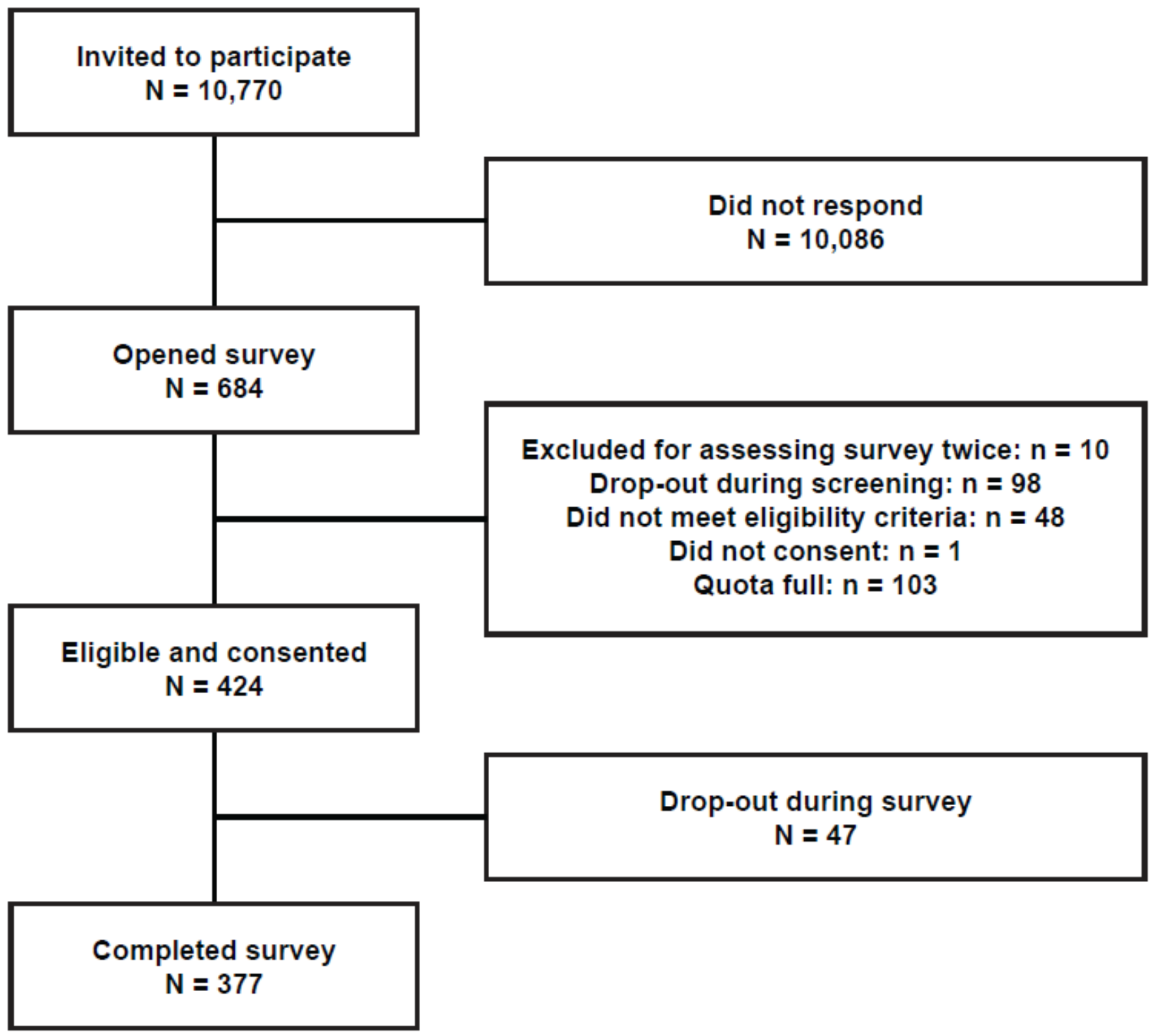

Supplemental Figure 3. Physician preferences from the discrete choice experiment by years of experience, linear-coded interacted multinomial logit (MNL) model (N = 377)

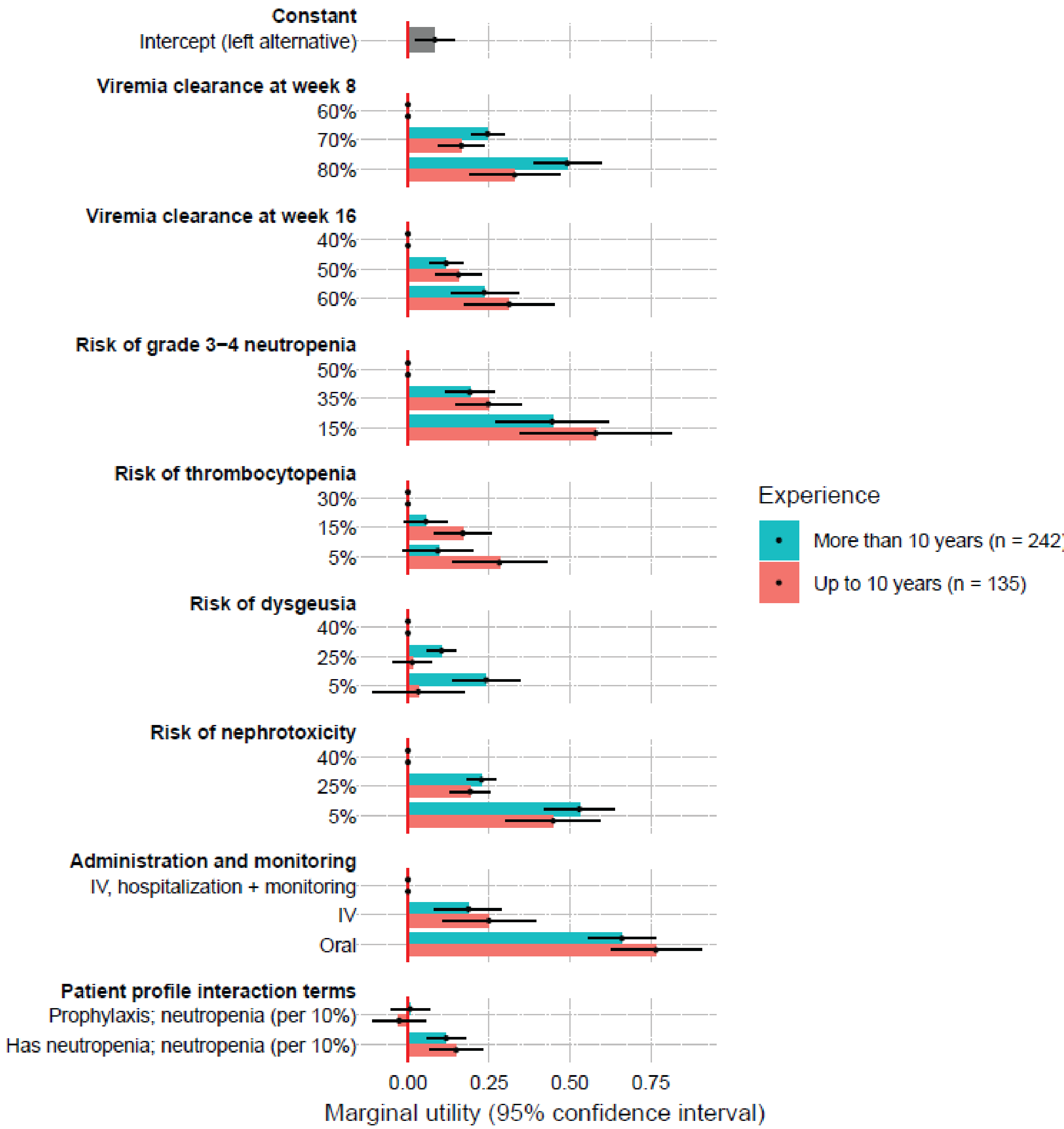

IV, intravenous.

Supplemental Figure 4. Physician preferences from the discrete choice experiment by specialization, linear-coded interacted multinomial logit (MNL) model (N = 377)

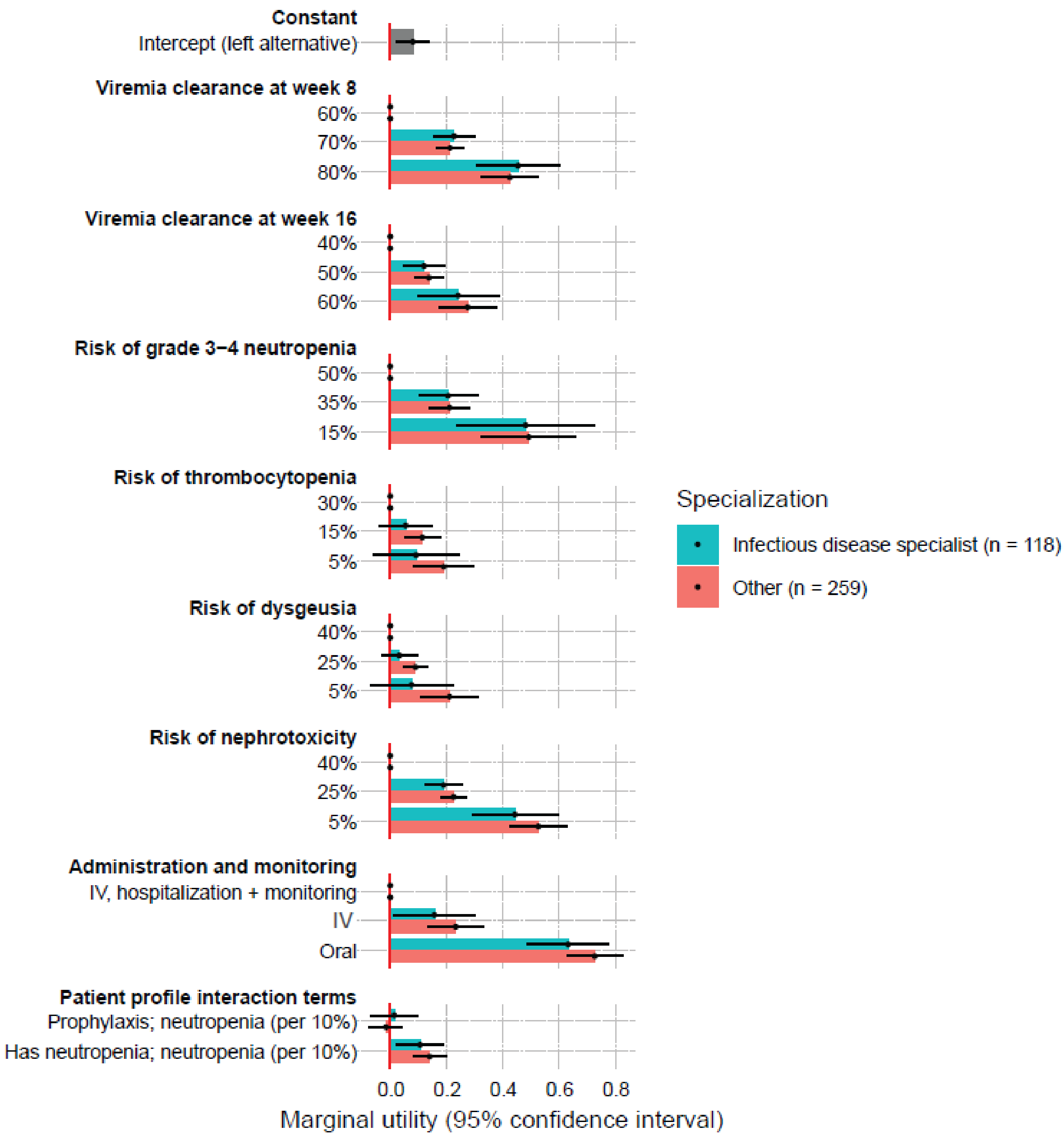

G, grade; IV, intravenous.

Supplemental Figure 5. Physician preferences from the discrete choice experiment by country, linear-coded interacted multinomial logit (MNL) model (N = 377)

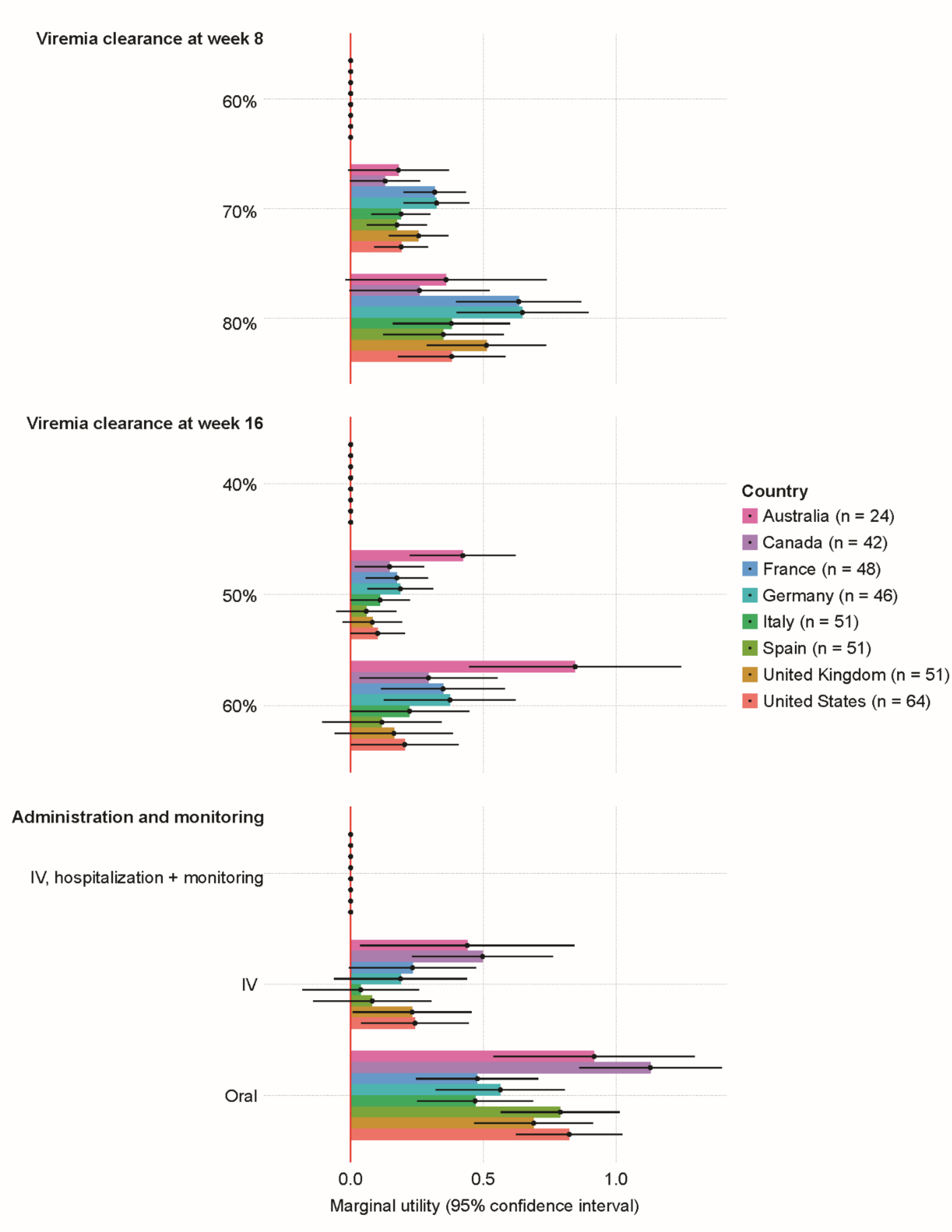

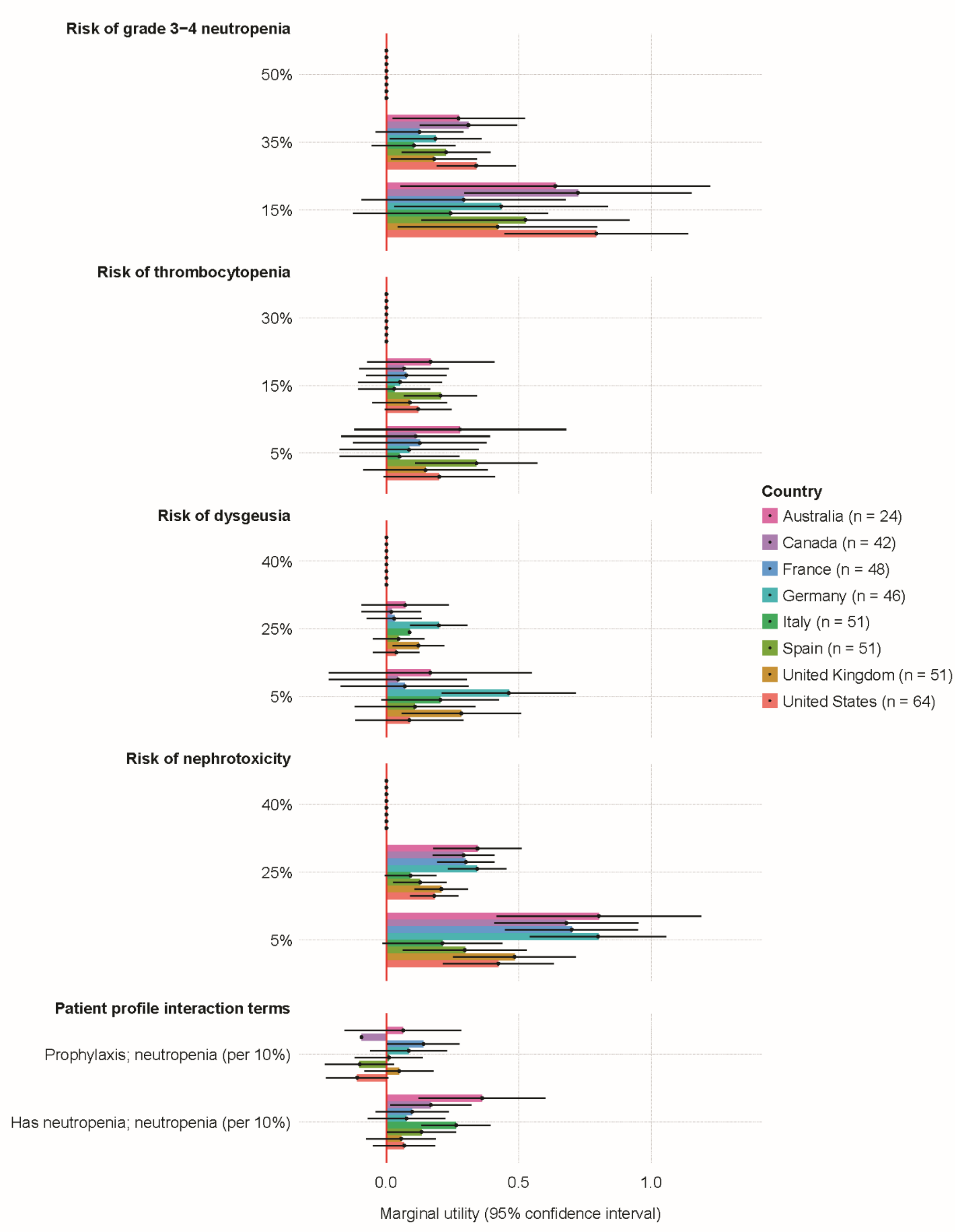

G, grade; IV, intravenous

Supplemental Figure 6. Probabilistic sensitivity analysis of attribute-level contributions to the benefit-risk score for maribavir and valganciclovir in patients with baseline absolute neutrophil count a) ≥1500/mm^3 (1.5×10^9/L) and b) <1500/mm^3 (1.5×10^9/L) in the multi-criteria decision analysis. The length of each bar is the product of the DCE-derived normalized attribute weight and standardized clinical trial data for the corresponding endpoint. Bootstrapping was employed for both types of uncertainty in this sensitivity analysis.

A

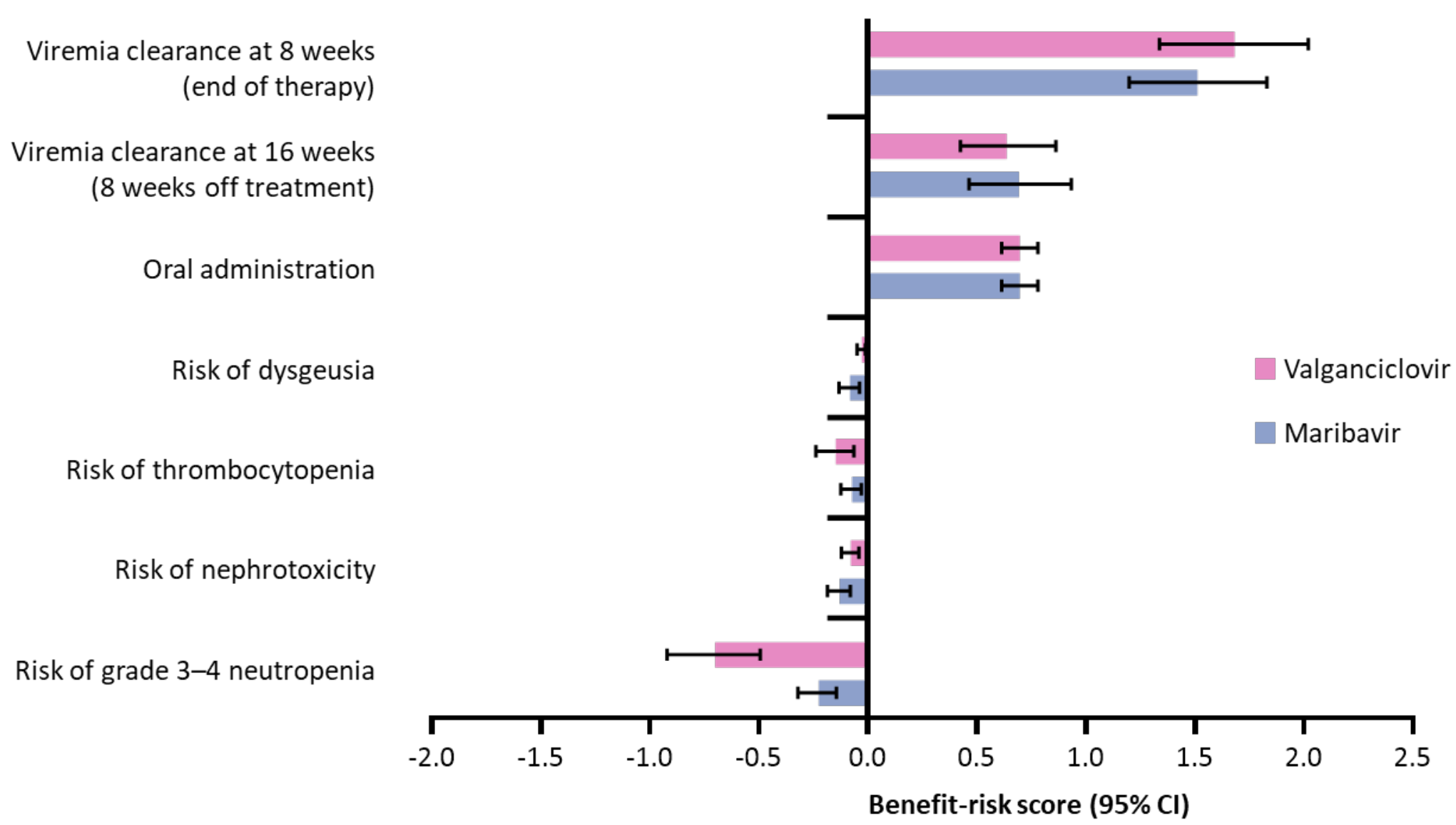

B

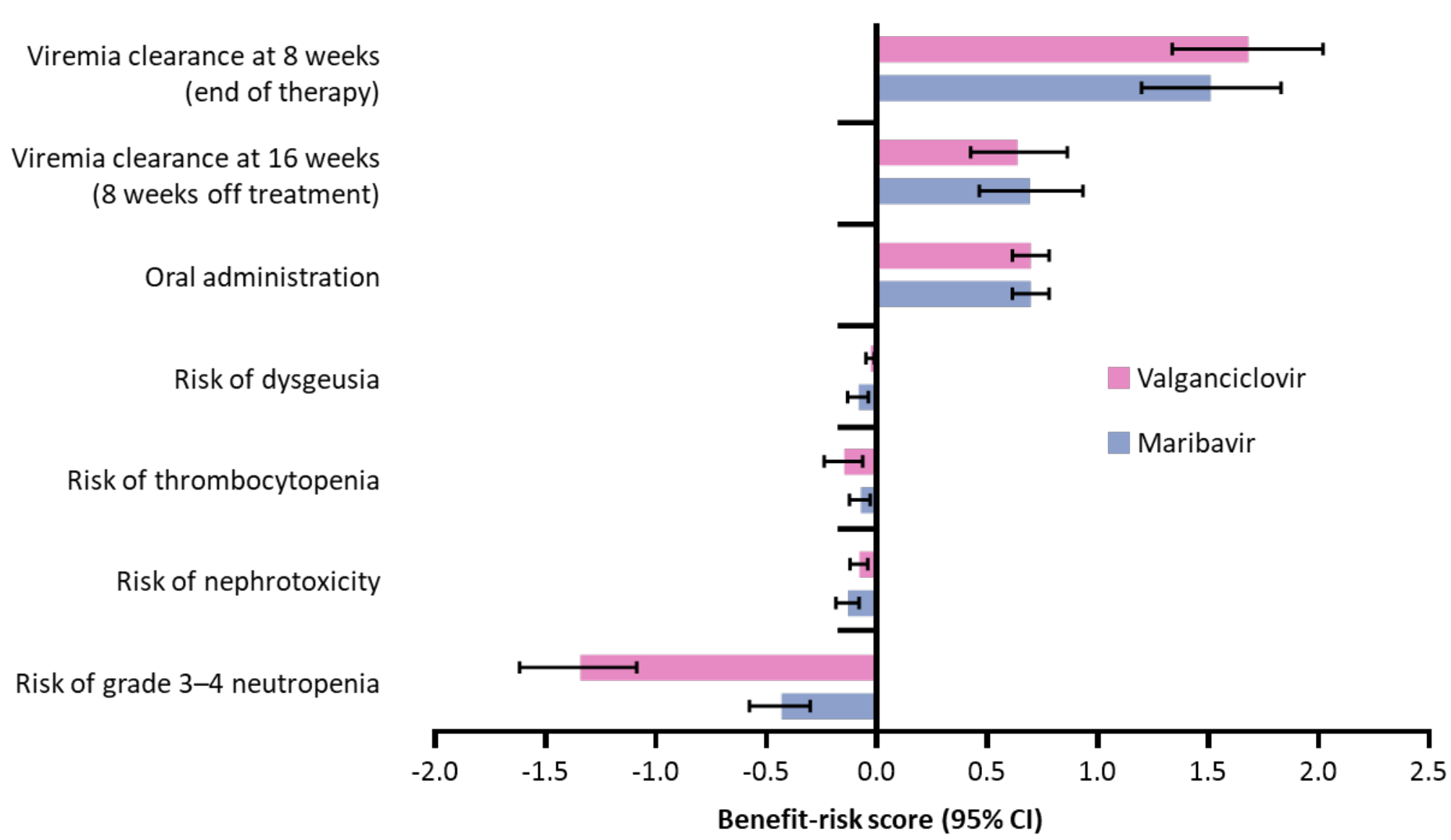

Supplemental Figure 7. Attribute-level contributions to the benefit-risk score for valganciclovir, maribavir, ganciclovir, and foscarnet in the multi-criteria decision analysis for patients with baseline absolute neutrophil count a) ≥1500/mm³ (1.5×10⁹/L) and b) <1500/mm³ (1.5×10⁹/L). The length of each bar is the product of the DCE-derived normalized attribute weight and standardized clinical trial data for the corresponding endpoint. Ganciclovir and foscarnet had a benefit-risk score of 0 for oral administration as these are intravenous agents.

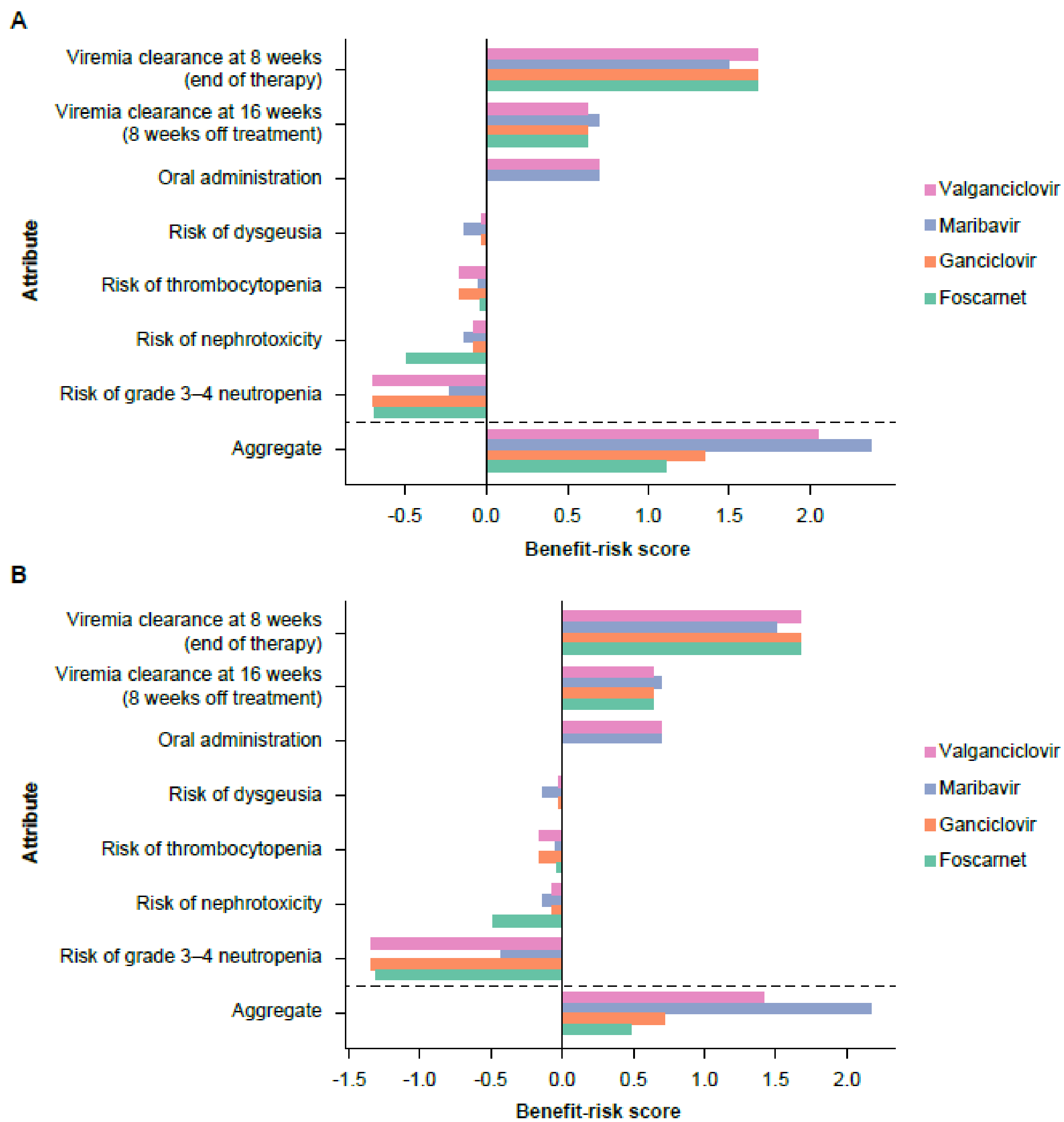

REFERENCES

1. Cho SY, Ar MC, Machado CM, et al. Epidemiology, treatment patterns, and disease burden of cytomegalovirus in hematopoietic cell transplant recipients in selected countries outside of Europe and North America: a systematic review. *Transpl Infect Dis.* 2023;25:e14083.

2. Moretti S, Zikos P, Van Lint MT, et al. Forscarnet *vs* ganciclovir for cytomegalovirus (CMV) antigenemia after allogeneic hemopoietic stem cell transplantation (HSCT): a randomised study. *Bone Marrow Transplant.* 1998;22:175-180.

3. Papanicolaou GA, Avery RK, Cordonnier C, et al. Treatment for first cytomegalovirus infection post-hematopoietic cell transplant in the AURORA Trial: a multicenter, double-blind, randomized, phase 3 trial comparing maribavir with valganciclovir. *Clin Infect Dis.* 2024;78:562-572.

4. Barkam C, Kamal H, Dammann E, et al. Improving safety of preemptive therapy with oral valganciclovir for cytomegalovirus infection after allogeneic hematopoietic stem cell transplantation. *Bone Marrow Res.* 2012;2012:874601.

5. Maertens J, Cordonnier C, Jaksch P, et al. Maribavir for preemptive treatment of cytomegalovirus reactivation. *N Engl J Med.* 2019;381:1136-1147.

6. Ruiz-Camps I, Len O, de la Cámara R, et al. Valganciclovir as pre-emptive therapy for cytomegalovirus infection in allogeneic haematopoietic stem cell transplant recipients. *Antivir Ther.* 2011;16:951-957.

7. Reusser P, Einsele H, Lee J, et al. Randomized multicenter trial of foscarnet versus ganciclovir for preemptive therapy of cytomegalovirus infection after allogeneic stem cell transplantation. *Blood.* 2002;99:1159-1164.

8. Inose R, Takahashi K, Takahashi M, et al. Long-term use of foscarnet is associated with an increased incidence of acute kidney injury in hematopoietic stem cell transplant patients: a retrospective observational study. *Transpl Infect Dis.* 2022;24:e13804.

9. Metafuni E, Chiusolo P, Sica S, et al. Foscarnet treatment of cytomegalovirus infection in haploidentical or unrelated donor transplants. *Bone Marrow Transplant.* 2018;53:1560-1567.

10. Zavras P, Su Y, Fang J, et al. Impact of preemptive therapy for cytomegalovirus on toxicities after allogeneic hematopoietic cell transplantation in clinical practice: a retrospective single-center cohort study. *Biol Blood Marrow Transplant.* 2020;26:1482-1491.

11. Janssen EM, Marshall DA, Hauber AB, Bridges JFP. Improving the quality of discrete-choice experiments in health: how can we assess validity and reliability? *Expert Rev Pharmacoecon Outcomes Res.* 2017;17:531-542.

12. Tervonen T, Schmidt-Ott T, Marsh K, Bridges JFP, Quaife M, Janssen E. Assessing rationality in discrete choice experiments in health: an investigation into the use of dominance tests. *Value Health.* 2018;21:1192-1197.

13. ChoiceMetrics Pty Ltd. *Ngene 1.1.1 User manual and reference guide.* Australia: ChoiceMetrics Pty Ltd; 2012.

14. Lancsar E, Fiebig DG, Hole AR. Discrete choice experiments: a guide to model specification, estimation and software. *Pharmacoeconomics.* 2017;35:697-716.

15. Manski CF. The structure of random utility models. *Theory and Decision.* 1977;8:229-254.

16. McFadden D. Conditional logit analysis of qualitative choice behavior. *IURD Working Paper Series* 1972; https://escholarship.org/uc/item/61s3q2xr. Accessed April 8, 2025.

17. Krinsky I, Robb AL. On approximating the statistical properties of elasticities. *Rev Econ Stat.* 1986;68:715-719.

18. Cordonnier C, Chevallier P, Verlinden A, Winston D, Gu J, Fournier M. Maribavir versus investigator-assigned therapy for refractory CMV infection (with/without resistance) in hematopoietic cell transplant recipients: subgroup safety analysis of a phase 3 study. 48th Annual Meeting of the European Society for Blood and Marrow Transplantation (EMBT); March 19-23, 2022; Prague, Czech Republic.

19. Takeda Pharmaceuticals America Inc. Livtencity. Prescribing information. 2024; https://content.takeda.com/?contenttype=pi&product=liv&language=eng&country=usa&documentnumber=1. Accessed March 25, 2025.

20. Genentech. Valcyte. Prescribing information. 2021; https://www.gene.com/download/pdf/valcyte_prescribing.pdf. Accessed March 25, 2025.

21. Clinigen Healthcare Ltd. Foscavir. Prescribing information. 2020; https://labeling.pfizer.com/ShowLabeling.aspx?id=8333. Accessed March 25, 2025.

22. Exela Pharma Sciences. Ganciclovir. Prescribing Information. 2017; https://www.accessdata.fda.gov/drugsatfda_docs/label/2017/209347lbl.pdf. Accessed July 8, 2025.

23. Asberg A, Humar A, Rollag H, et al. Oral valganciclovir is noninferior to intravenous ganciclovir for the treatment of cytomegalovirus disease in solid organ transplant recipients. *Am J Transplant.* 2007;7:2106-2113.

24. Avery RK, Alain S, Alexander BD, et al. Maribavir for refractory cytomegalovirus infections with or without resistance post-transplant: results from a phase 3 randomized clinical trial. *Clin Infect Dis.* 2022;75:690-701.
